# Community quorum sensing signalling and quenching: microbial granular biofilm assembly

**DOI:** 10.1038/npjbiofilms.2015.6

**Published:** 2015-05-27

**Authors:** Chuan Hao Tan, Kai Shyang Koh, Chao Xie, Joela Zhang, Xiao Hui Tan, Guo Ping Lee, Yan Zhou, Wun Jern Ng, Scott A Rice, Staffan Kjelleberg

**Affiliations:** 1 Singapore Centre on Environmental Life Sciences Engineering (SCELSE), Nanyang Technological University, Singapore; 2 Advanced Environmental Biotechnology Centre (AEBC), Nanyang Environment and Water Research Institute (NEWRI), Nanyang Technological University, Singapore; 3 The School of Civil and Environmental Engineering, Nanyang Technological University, Singapore; 4 The School of Biological Sciences, Nanyang Technological University, Singapore; 5 Centre for Marine Bio-Innovation and School of Biotechnology and Biomolecular Sciences, University of New South Wales, Sydney, Australia

## Abstract

**Background:**

Recent reports exploring the role of gradients of quorum sensing (QS) signals in functional activated sludge have raised the question of whether shared systems of signalling synthesis and degradation, or quorum quenching (QQ), across the community inform of the means by which QS biology regulate floccular and granular biofilm assembly.

**Aims:**

In this study, we aimed to explore the species origin and interactive role of QS and QQ activities in such highly diverse microbial biofilm communities.

**Methods:**

Here, such aims were addressed systematically by a comprehensive multi-pronged RNA-sequencing, microbiological and analytical chemistry experimental approach, using two related but independently evolved floccular and granular sludge communities.

**Results:**

Our data revealed a distinct difference between the QS and QQ potentials of the two communities, with different species largely displaying either QS or QQ functions. The floccular sludge community showed a high rate of QQ activity, and this rate was dependent on the acyl chain length demonstrating specificity of degradation. When the floccular biomass was transformed into the granular sludge, the QQ activity of the community was reduced by 30%. *N*-acyl homoserine lactones with four to eight carbons on the acyl chain accumulated at the granular stage, and their concentrations were at least threefold higher than those of the floccular stage. These findings corroborated meta-community analysis where a major shift in the dominant species from potential signal quenchers to producers was observed during the transition from flocs to granules, indicating the role of species composition and associated signalling activities in coordinating community behaviours.

**Conclusions:**

This study suggests that QQ has an important function in regulating community level QS signalling, and provides a mechanistic insight into the role of QS biology in complex community assembly.

## Introduction

In most natural and engineered ecosystems, bacteria predominantly reside in structured communities, in aggregations commonly known as microbial biofilms.^1^ These microbial consortia can be comprised of hundreds to thousands of bacterial species with varying metabolic capabilities and collectively displaying community level properties that are distinct from the planktonic cells.^2,3^ The overall composition and function of complex biofilms is typically driven by interactions between the different microbial species.^1,3^ Understanding the dynamics of interspecies interactions within such complex multi-species biofilms is challenging, yet important in order to control and regulate key ecosystem functions. These include, e.g., the engineering of highly stable and sustainable microbial communities for water and wastewater treatment, or the treatment of biofilm-related diseases.

In many cases, interspecies interactions involve communication via small diffusible signalling molecules, a mechanism generally termed quorum sensing (QS).^3^ Many bacteria use QS to synchronise population behaviours, including, but not limited to, biofilm formation, exoenzyme production and virulence factor secretion, to optimise population growth and survival in different environments.^4,5^ The *N*-acyl homoserine lactone (AHL)-mediated QS system is one of the most well-characterised bacterial communication systems and is present in ~10% of Proteobacteria isolated from various ecological niches.^6–8^ As a consequence of multiple community members sharing the same classes of signalling molecules, crosstalk or communication among bacteria of different species or even between organisms from different domains is possible.^9–12^ For example, *Burkholderia cepacia* can perceive the AHLs released by *Pseudomonas aeruginosa* in co-culture, leading to the formation of mixed microcolonies, in contrast to the formation of separate, non-mixed microcolonies in the absence of QS activity.^10^ AHLs are also present at biologically relevant concentrations in complex communities in a diverse range of habitats, including sputum from cystic fibrosis patients,^13^ microbial mats,^14^ the rumen ^15^ and wastewater treatment plants.^16,17^ These findings suggest that community level QS signalling is likely to be important across most habitats. Indeed, the role of AHL-mediated QS in the formation of complex, mixed-species sludge biofilms as well as the assembly of microbial granules was recently demonstrated in a membrane bioreactor^18^ and a sequencing batch reactor (SBR),^19^ respectively. For the SBR, it was found that the *in situ* production of specific AHLs was strongly and positively correlated with the morphogenesis of microbial granules. Elevated concentrations of AHLs were linked to the formation of granules, whereas decreased concentrations coincided with the disintegration of granules and conversion to the flocs.^19^ Most importantly, these processes were closely related to the abundance of a diverse range of microbial species in the granular communities, suggesting a highly complex, AHL-based community interaction.^19^ However, it is unclear how such interactions can be achieved and coordinated among the individual, phylogenetically different, species in complex communities.

One possible mechanism for the regulation of QS behaviour in the environment is via the control of signal concentrations, which is critical for effective QS signalling. It is possible that signal concentration in the environment could be regulated via specific enzymatic signal degradation activity or quorum quenching (QQ) by members of the community. Although QQ has been demonstrated in diverse microbial species,^20–23^ the ecological role and the impact of QQ on QS signalling remain largely unknown. Here we have explored the role of QQ as a regulator or modulator of community QS signalling. Using a complex floccular sludge commnity as a model, AHL-dependent QQ was found to be active and specific, mediated by community members of diverse origins. The assembly of floccular biomass into the granular sludge biofilms was inversely correlated with the competing QS and QQ activities of the community, suggesting an important function of QQ in coordinating community level QS signalling and behaviour.

## Materials and methods

### Bioreactor operation

An SBR (10×76.4 cm, diameter×height), seeded with floccular sludge community from a water reclamation plant (Ulu Pandan, Singapore), was operated at 22 °C for 82 weeks.^24^ The sludge culture was fed with synthetic wastewater (SWW) comprising 150 mg chemical oxygen demand per litre acetate and 50 mg chemical oxygen demand per litre propionate as carbon source, 20 mg/ l ammonium, 10 mg/l orthophosphate and trace nutrient elements.^25^ The bioreactor had a final working volume of 4 l. The bioreactor operation involved a 5-h cycle with two continuous stages of anaerobic, aerobic and anoxic periods. A total volume of 1 litre SWW was fed per cycle and resulted in a hydraulic retention time of 20 h. Bioreactor pH was maintained between 6.7 and 8.2 under the control of a programmable logic controller via dosing of either 9.12 g/l HCl or 10.0 g/l NaOH. System performance of the bioreactor was monitored via weekly cycle studies. Concentrations of ammonium, nitrite, nitrate and orthophosphate, as well as concentrations of sludge biomass, i.e., mixed liquor-suspended solids and mixed liquor volatile-suspended solids were determined in accordance to the American Public Health Association (APHA) standard engineering methods.^26^ One millilitre aliquots of sludge samples were collected at the end of the anoxic period. The sludge biomass was immediately harvested after centrifugation (5 min, 8000*g,* 4 °C) and stored at −80 °C for subsequent RNA analysis. Similarly, 50 ml aliquots of treated effluents were kept at −80 °C immediately after sampling for later AHL analysis. At weeks 40–44, a portion of the floccular sludge culture from the SBR was collected and used to seed a second SBR (6×200 cm, diameter×height). The second SBR was operated according to the first SBR, with some modifications, to facilitate the formation of microbial granules (as previously described in detail).^19^ Briefly, the second SBR was operated at an hydraulic retention time of 12 h and the settling time for each cycle was reduced from 60 to 5 min within the first 5 weeks of reactor operation.^19^ Here granules were defined as compact aggregates with a minimum particle diameter of 100 μm and a sludge volumetric index (SVI_5_, a measure of biomass density/compactness) of 50 ml/g or less,^19,27^ while flocs were loosely aggregated biomass with particle diameter of 100 μm or less and a SVI_5_ of at least 50 ml/g.

### Bacterial strains and growth conditions

AHL biosensors including *Agrobacterium tumefaciens* A136,^28^ *Chromobacterium violaceum* CV026 (ref. 29) and *Escherichia coli* pJBA357,^30^ as well as *P. aeruginosa* MH602 (ref. 31) and *E. coli* JM109 (ref. 32) were routinely maintained in Luria-Bertani medium. The following antibiotics were supplemented into the growth medium whenever necessary: tetracycline (4.5 μg/ml), spectinomycin (50 μg/ml), kanamycin (50 μg/ml), ampicillin (100 μg/ml) or gentamycin (40 μg/ml). The genotypes of these bacterial strains are listed in Supplementary Table S1. Cultures were incubated at 30 °C for 24–48 h.

### *N*-Acyl homoserine lactones

Synthetic AHLs (>97%) were purchased from Sigma-Aldrich (Singapore). Each AHL was expressed as an acronym: C4-HSL (*N*-butyryl-DL-homoserine lactone), C6-HSL (*N*-hexanoyl-DL-homoserine lactone), 3OC6-HSL (*N*-(3-oxohexanoyl)-DL-homoserine lactone), C7-HSL (*N*-heptanoyl-DL-homoserine lactone), C8-HSL (*N*-octanoyl-DL-homoserine lactone), 3OC8-HSL (*N*-(3-oxooctanoyl)-l-homoserine lactone), C10-HSL (*N*-decanoyl-DL-homoserine lactone), 3OC10-HSL (*N*-(3-oxodecanoyl)-L-homoserine lactone), C12-HSL (*N*-dodecanoyl-DL-homoserine lactone), 3OC12-HSL (*N*-(3-oxododecanoyl)-L-homoserine lactone), 3OHC12-HSL (*N*-(3-hydroxydodecanoyl)-DL-homoserine lactone), C14-HSL (*N*-tetradecanoyl-DL-homoserine lactone) and 3OC14-HSL (*N*-(3-oxotetradecanoyl)-L-homoserine lactone).

### Determination of AHL inactivation kinetics

Synthetic AHLs dissolved in dimethyl sulphoxide (Sigma-Aldrich) were added individually or in combination with the sludge culture microcosms (harvested from the bioreactor) at 5 μM for each AHL, and incubated at 22 °C with constant shaking (200 r.p.m.). Samples were collected from each microcosm at different time points, and the sludge biomass was removed by centrifugation (10 min, 8000*g*). Residual AHLs in the sludge supernatant were extracted and quantified by high-performance liquid chromatography–tandem mass spectrometer (Shimadzu, Singapore) as described below. Heat-inactivated sludge and SWW controls were included. The adsorption of AHLs to the sludge biomass was determined by comparing the residual AHLs in the SWW and the heat-inactivated sludge controls, immediately after addition, as well as after 1 h incubation. The pH of each sludge microcosm (bulk liquid) was monitored throughout the study and ranged between 6.7 and 6.9. The impact of pH on the integrity of AHLs was also assessed by adding synthetic AHLs to SWW buffered at different pH values ranging from 6.7 to 8.3. Linear and nonlinear regression curves were modelled using Prism (GraphPad, San Diego, CA, USA ). Signal half-life was estimated based on zero and first-order kinetics.^33^

### Detection and quantification of AHLs by high-performance liquid chromatography–tandem mass spectrometry (LC-MS/MS)

Detection and quantification of AHLs from bioreactor-treated effluents or sludge supernatants were conducted as described by Tan *et al.*^19^ Briefly, AHLs were extracted and concentrated from the supernatants using dichloromethane (Sigma-Aldrich). The dichloromethane extracts were analysed and quantified using high-performance liquid chromatography–tandem mass spectrometer system (Shimadzu LCMS8030, Shimadzu). All samples were chromatographed by high-performance liquid chromatography (Shim-pack XR-ODS C18 column) at a flow rate of 0.3 ml/min. The mobile phase consisted of a linear gradient (40–95%) of solvent B (methanol with 0.1% formic acid) and solvent A (25 mM ammonium formate with 0.1% formic acid). Effluents were ionised by electrospray ionisation under positive mode and detected using the multiple reaction monitoring approach.^14,34^ Matrix-matched multiple reaction monitoring experiments were conducted for all AHLs.^19^ Each standard multiple reaction monitoring profile, including specific liquid chromatography (LC) retention time, appearance of precursor ion *m/z* and two transition ions, as well as relative intensity of the two transition ions, was used as a reference. To confirm the identity of the putative AHLs, a full scan ranging from *m/z* 100 to 350, coupled with precursor ion scan mode, was conducted in comparison with the standard AHLs.^14^ For AHL quantification, matrix-matched standard curves, ranging from 0.5 to 200 μg/l, were constructed. Two transition ions, characteristic of the respective AHLs, were used to identify the AHL, and the transition ion with highest intensity was used to construct the standard curves. The analyte peak areas were integrated using LabSolutions (Shimadzu). The limits of detection and quantification for each AHL were calculated with a signal-to-noise ratio of 3.3 and 10, respectively.^14^ The amount of each putative AHL in the sample was calculated based on the standard curve and the extraction efficiency of the respective AHL. The extraction efficiency for each AHL was calculated based on the recovery of the standard AHLs added at 5 and 50 μg/l to the heat-inactivated sludge supernatant sample matrix. Blank injections were performed at the intervals of sample injections to avoid sample carryover.

### Isolation of sludge community members

A total of five independent isolation experiments were performed from weeks 40 to 44 of the bioreactor operation. Each time, 20 ml of uniformly mixed sludge samples were collected from the bioreactor at the end of an operational cycle. Sludge samples were vortexed vigorously for 5 min to disperse the floccular cells, serially diluted and spread onto the following culture media: nutrient broth, R2A, *Pseudomonas* isolation, SWW and ABT^35^ supplemented with 1.5% (w/v) agar each. The plates were incubated at 22 °C for 5 days. A total of 330 strains with distinct colony morphologies were isolated and identified by ribosomal DNA (rDNA) gene sequencing.

### Identification of isolates

The universal primers 27F (5′-AGAGTTTGATCMTGGCTCAG-3′) and 1492R (5′-ACGGTTACCTTGTTACGACTT-3′) targeting the 16S rDNA gene were used to sequence bacteria,^36^ while primers ITS1 (5′-TCCGTAGGTGAACCTGCGG-3′) and ITS4 (5′-TCCTCCGCTTATTGATATGC-3′) were used to sequence the internal transcribed spacer region to identify fungi.^37^ Sequencing was performed using the Big Dye Terminator (Applied Biosystems, Singapore). The sequences were assembled using DNA Baser sequence assembler v3.5.2 (Heracle Biosoft, www.DnaBaser.com) and matched using online sequence similarity search tools, such as BLAST, Greengenes and SILVA. The isolate sequences obtained in this study are available via GenBank under accession numbers KC252636–KC252965.

### Screening of AHL producers

For the *A. tumefaciens* A136 bioassay, ABT indicator agar^35^ with 50 μg/ml of 5-bromo-4-chloro-3-indolyl-b-D-galactopyranoside (X-gal) was prepared, while Luria-Bertani agar was used for the *C. violaceum* CV026 bioassay. Ten microlitres of overnight cultures of biosensors were spotted onto the indicator plates, while 10 μl overnight cultures of isolates were placed 5 mm apart from the biosensor spot and incubated at 30 °C for 48 h. AHLs produced *in situ* were detected using *E. coli* JBA357 biosensor. Briefly, 100 μl of 5× diluted *E. coli* JBA357 overnight culture were added to 100 μl of samples in a 96-well plate and incubated at 22 °C with constant shaking at 200 r.p.m. for 4 h before examination by confocal laser scanning microscope (Zeiss LSM 710, Carl Zeiss Pte. Ltd., Singapore) at an excitation/emission wavelength of 488/522–535 nm. *E. coli* JM109, *P. aeruginosa* MH602 and synthetic AHLs were included as controls.

### Screening of AHL quenchers

AHLs (3OC6-HSL, 3OC8-HSL and 3OC12-HSL) were added individually to the overnight cultures of isolates at 5 μM, and incubated at 22 °C with constant shaking at 200 r.p.m. for 2 h. After centrifugation and ultraviolet sterilisation, residual AHLs were quantified using the *A. tumefaciens* A136 bioassay.^38,39^ Briefly, six agar bars with a width of 1 cm each were aseptically carved from the ABT indicator plates. Five microlitres of samples were spotted at one end of the agar bars. Approximately 0.5 μl of overnight culture of the *A. tumefaciens* A136 was spotted at progressively further distances from the loaded samples. The plates were incubated at 30 °C for 24 h. AHLs added to Luria-Bertani at pH 6.7, 7.2 and 10.2, and *E. coli* JM109 cultures were included as controls. The pH for all overnight cultures were determined to be <7.3 by the end of the experiments.

### Phylogenetic analysis

The 16S rDNA sequences were aligned by ClustalW Multiple alignment package (http://www.mbio.ncsu.edu/bioedit/bioedit.html) and a consensus region covering all the sequences was selected for further analysis. The aligned sequences were subjected to phylogenetic tree construction using the neighbour-joining method^40^ provided by the MEGA4 (http://www.megasoftware.net/mega4/mega.html). Maximum likelihood bootstrap analyses were carried out with 1,000 replicates.

### RNA-sequencing analysis

Total RNA was extracted from sludge using FastRNA Pro Soil-Direct kit (MP Biomedicals, Singapore) according to the manufacturer’s guidelines. Extracted RNA was subjected to DNA removal using the TURBO DNA-free kit (Applied Biosystems). Total RNA, 200 ng, was used for complimentary DNA library preparation according to the manufacturer’s instructions (Illumina, Singapore). Each complimentary DNA library was ligated with a unique adaptor sequence for sample multiplexing. A total of 16 different complimentary DNA libraries were pooled and sequenced by MiSeq System (Illumina). The RNA-sequencing data were analysed using a fast tag-based method as described.^19^ Briefly, a universal primer (5′-CGACRRCCATGCANCACCT-3′) for the 16S rRNA hypervariable region (V6) was used to scan each sequencing read to obtain all 33 nucleotide tag sequences downstream of the primer, and the 33 nucleotide sequences are defined as the V6 tag of the 16S rRNA gene. The universal primer matches 94% of 16S rRNA sequences in the RDP database.^41^ To eliminate sequencing error-derived V6 tags, only V6 tags that were observed in at least two different reads were kept for analysis. In addition, V6 tags were removed when any single type of sequencing read accounted for ⩾50% of all reads covering that V6 tag. This was done to eliminate sequencing artefacts where identical duplicated reads were generated from single templates.

### Statistical analysis

All statistical analyses were conducted using Prism (GraphPad) or R (www.r-project.org). Holm–Sidak’s multiple comparison tests were performed to compare the half-lives of AHLs in different samples or to examine AHL expression between different sludge samples. Pearson correlation coefficients were determined, and false discovery rate corrections were made for all multiple correlations. The correlation matrixes were clustered using unsupervised hierarchical clustering method based on Euclidean distance with complete linkage as coded by Cluster 3.0 and visualised using Java TreeView (Open Source Clustering Software, Tokyo, Japan).

## Results

### The floccular sludge community degrades AHLs differentially based on acyl chain length

**Figure 1:**
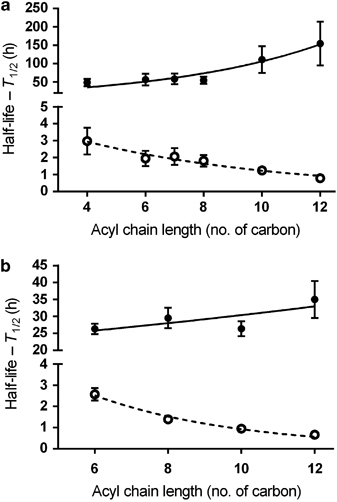
Degradation of *N*-acyl homoserine lactones (AHLs) based on the function of acyl chain length in the floccular sludge. The degradation value is expressed as the signal half-life. A mixture of unsubstituted (**a**) and oxo-substituted (**b**) AHLs was added to the live (open circle) or the heat-inactivated (solid circle) sludge to a final concentration of 5 μM for each AHL. The sludge mixture was incubated at room temperature with constant shaking at 200 r.p.m. The pH of each sludge mixture (bulk liquid) was monitored throughout the study and ranged between pH 6.7 and 6.9. Residual AHLs were extracted and quantified using LC-MS/MS at different time points. The signal half-life for each AHL was estimated based on the zero-order or first-order kinetics. Error bars are defined as s.e.m. (*n*=3, biological replicates). Holm–Sidak multiple *t*-tests were performed (live versus heat-inactivated control for each AHL) and corrected *P* values (*P*<0.05 for all pairs of comparison) are reported.

A highly diverse floccular sludge community bioreactor undergoing simultaneous nitrification, denitrification and phosphorus removal was used as the experimental system (Supplementary Figure S1). To determine whether AHL-specific QQ activity was present in this model system, synthetic AHLs were added individually or as a mixture to the floccular sludge to characterise the rates of signal degradation. Approximately 10–30% of the AHLs were lost due to adsorption to the biomass, while loss of the remaining 70–90% of signal was attributed to biological activity (Supplementary Figure S2). This was evident by the substantially shorter AHL half-lives in the presence of live sludge than when incubated in the presence of heat-inactivated sludge (*P*<0.05 for all AHLs; Figure 1). In the presence of live sludge, the signal half-life was inversely proportional to the length of the acyl chain, regardless of the substitution at the C3 position (Figure 1). For example, the half-lives for short-chain signals C6-HSL and 3OC6-HSL were 1.95±0.45 and 2.57±0.29 h, respectively, whereas the half-lives of the long-chain signals C12-HSL and 3OC12-HSL were considerably shorter at 0.79±0.08 and 0.66±0.06 h, respectively. The pH of the live sludge microcosm was consistent at 6.7–6.9, and the data for the SWW-buffered controls indicated that there was little spontaneous degradation of AHLs between pH values of 6.7–8.3 (Supplementary Figure S3). Therefore, it is likely that the loss of AHLs observed here was due to active enzymatic digestion rather than passive, chemical degradation.

### A significant proportion of the floccular sludge community members either produce or quench AHLs

**Figure 2:**
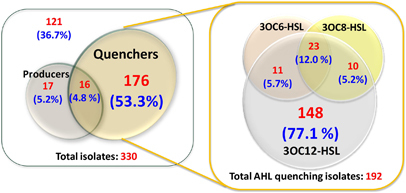
Distribution of isolates according to their capacity to produce and/or quench *N*-acyl homoserine lactones (AHLs). A total of 330 isolates, including 307 and 23 strains of bacterial and fungal origins, respectively, were cultured from the floccular sludge community during the bioreactor operation from weeks 40–44. All isolates were screened for AHL production by different bioassays and LC-MS/MS, as well as for their capability to quench 5 μM of AHLs with short (3OC6-HSL), medium (3OC8-HSL) and long (3OC12-HSL) acyl chains after 2 h incubation. Residual AHLs were quantified using the *A. tumefaciens* A136 agar-based spot bioassay.

To gain a further insight into the relationships between the specificity of QQ activity and signal production in the floccular sludge community, 307 strains of bacteria and 23 strains of fungi, corresponding to 50 bacterial and four fungal genera were isolated and tested for their capability to produce or degrade AHLs (Supplementary Table S2). The bacterial isolates represented 3.5% of the total community members identified by RNA-sequencing (Supplementary Tables S3 and 4). AHL production was primarily assessed based on activation of different biosensors, including *E. coli* JBA357,^30^ *A. tumefaciens* A136^28^ and *C. violaceum* CV026.^29^ The AHL profile of each putative signal producer was subsequently determined using LC-MS/MS. AHL degradation was assayed using AHLs with short (3OC6-HSL), medium (3OC8-HSL) and long (3OC12-HSL) acyl chains. A large proportion of floccular sludge isolates, 65%, were either AHL signal producers or quenchers (Figure 2). Although only ~10% of the isolates could produce AHLs, as many as 58.1% of the isolates were able to quench at least one of the AHLs examined. A relatively small proportion of the isolates, 4.8%, both produced and quenched AHLs. The remaining 36.7% of isolates neither produced nor quenched AHLs. All of the AHL quenchers were capable of inactivating the long-chain AHL, 3OC12-HSL. Although 77% of the quenchers could only degrade the long-chain AHLs, ~23% could degrade long-chain AHLs as well as short or medium-chain AHLs.

### AHL quenching community members are phylogenetically diverse and distinct from AHL producers

**Figure 3:**
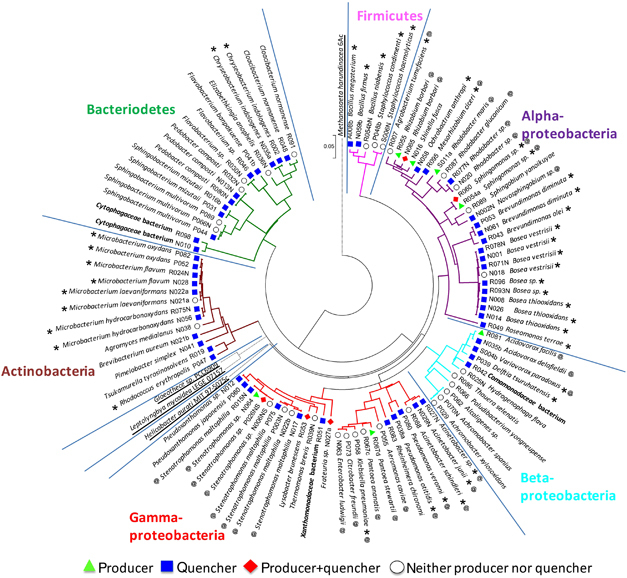
Evolutionary relationship of 100 representative bacterial isolates (taxa) based on the 16S rDNA gene sequences. Each representative was selected for their uniqueness in terms of *N*-acyl homoserine lactone (AHL) production and/or quenching properties. The evolutionary relationship was inferred using the neighbour-joining method. The bootstrap consensus tree inferred from 1,000 replicates was taken to represent the evolutionary relationship of the taxa analysed. Branches corresponding to partitions reproduced in <50% bootstrap replicates were collapsed. The tree was drawn to scale, with branch lengths reflecting evolutionary distances. The evolutionary distances were computed using the maximum composite likelihood method and are in the units of the number of base substitutions per site. All positions containing gaps and missing data were eliminated from the data set (complete deletion option). There were a total of 1,031 positions in the final data set. Phylogenetic analyses were conducted in MEGA4. The outgroups, retrieved from the GenBank database, are underlined. The isolated strains are classified as producer (green triangle), quencher (blue square), producer and quencher (red diamond) and neither producer nor quencher (open circle). The genera in which AHL production (@) or AHL degradation (*) activities have been reported in the literature are indicated. The closest relative with at least 97% sequence identity for each strain, except for those strains highlighted in bold, is shown.

The evolutionary relationships among the bacterial isolates, based on their 16S rDNA sequences, indicated that there was no specific clade associated with QQ activity, while the AHL producers were all classified as alpha, beta or gamma Proteobacteria (Figure 3). These QS isolates represented genera that have previously been reported to be AHL producers, such as *Acidovorax*, *Pantoea*, *Rhizobium*, *Rhodobacter*, *Sphingomonas* and *Stenotrophomonas,* as well as novel genera that have not been reported to synthesise AHLs, including *Frateuria*, *Lysobacter* and *Shinella* (Figure 3). The LC-MS/MS analysis further revealed that the isolates produced AHLs of various acyl chain lengths ranging from 4 to 14 carbons (Supplementary Table S5). Every signal producing isolate synthesised more than one type of AHL and multiple genera/species could produce the same AHL (Supplementary Tables S2 and 5). For example, isolates from six out of nine bacterial genera were capable of synthesising 3OC8-HSL (Supplementary Table S5), the dominant signal detected in the sludge community *in situ* (Supplementary Figure S4). In contrast, the AHL quenchers were classified into four different bacterial phyla, including the Proteobacteria, Actinobacteria, Bacteriodetes and Firmicutes, as well representatives from the Fungi (Figure 3 and Supplementary Table S2). Although some of the QQ isolates represented genera that are commonly reported to degrade AHLs, such as *Ochrobacterium*, *Variovorax*, *Bacillus* and *Rhodoccus*, most of the QQ isolates identified here have not previously been shown to have this activity. These novel QQ organisms were distributed throughout the genera *Novosphingobium, Acidovorax, Rheinheimera, Tsukamurella, Flavobacterium* and *Candida*, among others. Interestingly, some of the isolates were capable of both synthesising and degrading AHLs, e.g., *Rhizobium borbori* N065. In addition, there was considerable variation in function even at the species level. For example, 10 strains of *Stenotrophomonas* sp. with identical 16S rDNA sequences were able to produce AHLs, while the remaining six strains were not (Supplementary Table S2). Likewise, not every strain of the same species was able to quench AHLs, as was the case for *Stenotrophomonas maltophilia*. The specific QS and QQ characteristics of each isolate implies that AHL signalling at the community level is complex, and varying QS and QQ species composition may have different impacts on various signalling-regulated community behaviours.

### Community shift from signal quenchers to producers may lead to signal accumulation and granular biofilm assembly

**Figure 4:**
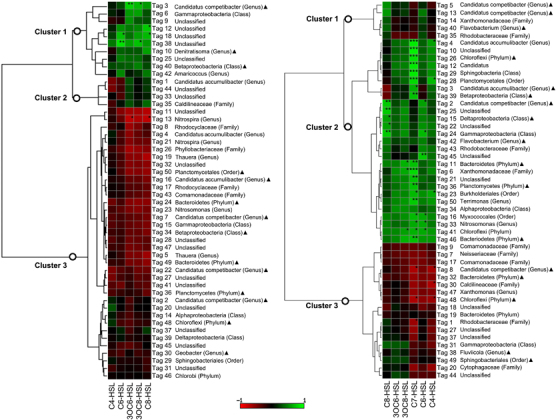
A comparison between the signalling properties of the floccular (left panel) and the granular (right panel) sludge communities. The relationships between the abundance of the top 50 most dominant community members (each represented by a unique V6 tag) and the concentration profile of *N*-acyl homoserine lactones (AHLs) over time were calculated based on Pearson correlation for the respective floccular and granular sludge communities. The Pearson correlation coefficient is colour coded, indicating relationships from strongly positive (+1, green) to strongly negative (−1, red) interactions. ^▲^Unclassified based on the taxonomy classification pipeline stated in Materials and methods section. The closest relative with sequence identity matching >97% is shown where it is applicable. False discovery rate corrections for multiple comparisons were performed, and significant differences are indicated as follows: *P<0.05, **P<0.01 and ***P<0.001. The signalling properties of the granular sludge community was adapted from Tan *et al.*^19^

To explore the role of species composition and signalling on community function, in terms of biofilm structure, the signalling properties of two independent communities, i.e., the floccular and the granular sludge communities^19^ were compared. Both floccular and granular sludge bioreactors were first seeded with the simultaneous nitrification, denitrification and phosphorus removal floccular sludge inoculum,^24^ and were either operated to maintain the floccular sludge or the conditions were changed to facilitate the formation of microbial granules.^19^ The species composition and the signal profile of both communities were characterised over time. Pearson correlation analysis of the top 50 most abundant floccular sludge community members (including non-cultivable members by RNA-sequencing) and the concentration of individual AHLs *in situ* revealed the dominance of community members that were negatively correlated with AHL production over 82 weeks (Figure 4, left panel, Cluster 3). Approximately 72% of the Pearson relationships were classified as negative, whereas only 20% were positive at the floccular stage (Figure 4, left panel). The high percentage of negative relationships was consistent with the higher proportions of QQ isolates in the floccular sludge community (Figures 2 and 3). When flocs were transformed into granules, there was a significant decrease in the negative correlation between community members and AHL concentration, from 72 to 38%, concomitant with an increase from 20 to 54% of positive relationships (Figure 4, right panel).^19^ These findings thus indicate a distinct community shift from potential signal quenchers to producers during the transition from flocs to granules. Accordingly, the QQ activity of the granular sludge was substantially reduced relative to the floccular sludge (Supplementary Figure S5). For example, the half-life of 3OC8-HSL was ~28% longer for the granular sludge compared with the floccular sludge. In addition, AHLs, including 3OC6-HSL, 3OC8-HSL, C6-HSL and C8-HSL, accumulated to at least a threefold higher concentration during the granular stage compared with the floccular stage (*P*<0.05 for all AHLs) (Supplementary Figure S6). In particular, the dominant signal 3OC8-HSL increased from an average of 25 pmol/g at the floccular stage to 85 pmol/g at the granular stage (*P*<0.016). The elevated amount of AHLs was previously associated with the increased expression of extracellular polymeric substances and the formation of microbial granules, whereas decreased AHL levels were linked to granular dispersal and conversion to floccular biomass.^19^ Here it appears that signal gradients could be regulated by the QQ activity of the community depending on the species composition and the associated QQ capacity. It is therefore possible that the balance between the competing QS and QQ activities may determine the community signalling behaviour or function, e.g., the assembly of microbial granular biofilms.^19^

## Discussion

The study of interspecies interactions is important for developing a systematic understanding of the physiology of high-density, matrix-encased microbial communities, which are now widely appreciated to be the dominant mode of growth for bacteria in natural and engineered ecosystems.^3,42^ Communication via diffusible signalling molecules has been proposed to be one of the important interactions among species of diverse origins,^3^ and there is growing evidence that complex communities in natural environments produce QS signals *in situ* to regulate different community behaviours.^14,16,18,19^ Although these findings have demonstrated the roles of QS in complex communities, the question of how competing activities, such as the QQ modulation of QS in complex communities, are mediated remains to be addressed. Using microbial granular assembly as a model for QS-mediated community behaviour,^19^ this study has shown that QQ strongly suppresses QS signal accumulation in the floccular sludge (Figure 1), and may thus inhibit the formation of microbial granules. When the QQ suppression was partially relieved, indicated by the increased signal half-lives at the granular stage (Supplementary Figure S5), the signal concentrations were increased significantly (Supplementary Figure S6), suggesting that QQ can be an effective modulator of community QS signalling. Indeed, addition of exogenous QQ enzymes to a membrane bioreactor can delay mixed-species biofilm development on membrane surfaces.^18,43^ In our study, we further established that QQ is an inherent function of complex communities that may have a strong influence on many interspecies interactions based on AHL signalling. Therefore, in contrast to some natural habitats where environmental factors have a dominant role in regulating the levels of QS signals, such as photosynthesis-driven cycles of high pH (up to 9.4), which strongly attenuates AHL signalling in microbial mats,^14^ we found that pH (up to <8.3) and physical signal adsorption were less important than enzymatic activity in the inactivation of signals in our model system (Supplementary Figures S2 and 3). This therefore suggests that QQ activity was the primary mechanism of signal removal, and hence serves as a key regulator of signal levels at different stages of granulation life cycle.

Based on the results obtained in this study, we submit that the overall AHL accumulation in the granulation system was directly related to the relative proportions and the activities of QQ and QS organisms in the community. As many as 60% of isolates from the floccular sludge community, representing 32 bacterial and 4 fungal genera were signal quenchers, while only 10% of the isolates (9 bacterial genera) produced AHLs (Figures 2 and 3). Whereas the proportion of isolated AHL producers observed here is consistent with observations from other environments,^7,8^ the frequency of AHL quenchers is considerably higher than reported in other studies (~5–15% of isolates).^7,8^ The higher proportion of AHL quenchers detected here is likely a consequence of testing for QQ activity using a range of AHLs as targets, including the long-chain AHLs, which have not typically been used to detect QQ activity. Interestingly, AHLs were degraded in a chain length–dependent manner *in situ* where the long-chain AHLs were preferentially inactivated (Figure 1), which is consistent with the observations of a higher proportion of isolates being able to degrade long-chain AHLs (100% of AHL quenchers) as compared with short- and medium-chain AHLs (~23% of AHL quenchers; Figure 2). Thus, the QQ activities of the spectrum of isolates collected were more generally reflected in the types of signals that were degraded by the whole community *in situ*. This observation also corresponded with the profile of AHLs that were identified in the floccular sludge community, where only AHLs with short or medium acyl chains were detected at low concentrations, while signals with long acyl chains ⩾10 carbons were not detected at all (Supplementary Figure S4). Almost every QS isolate produced at least one long-chain AHL (Supplementary Table S5), suggesting that the sludge community has the capacity to synthesise the long-chain signals *in situ*. Thus, the lack of long-chain AHLs in the sludge was not due to the absence of producer strains, and is most likely a consequence of specific QQ activity by the community.

The isolation studies indicated that the floccular sludge community was dominated by the signal quenchers (60%; Figure 2), and this was also reflected in the whole-community analysis where >70% of the top 50 most abundant community members were negatively correlated with AHL accumulation (Figure 4; left panel—Cluster 3). Although many factors may have contributed to the negative relationships, one key factor could be that Cluster 3 community members were potential quenchers that antagonise signal accumulation. In support of this, one of the cluster members, Tag 43 (*Diaphorobacter nitroreducens* R042), was isolated and confirmed to be an AHL quencher (Supplementary Table S2). The higher proportions of signal quenchers relative to the producers in the whole community may thus explain the short half-lives of AHLs, and hence the low AHL concentrations at the floccular stage (Figure 1 and Supplementary Figure S6). Conversely, there was a shift in the community towards QS, i.e., positive correlations, with a concomitant decrease in QQ organisms, i.e., negative correlations, when the biomass was transformed from flocs to granules (Figure 4, right panel). For example, *Lysobacter brunescens* R037 (Figure 4, right panel—Tag 6 in granular sludge), a signal producer that has been isolated from the community, was almost undetectable at the floccular stage but was highly abundant during granulation.^19^ In contrast, the signal quencher, *D. nitroreducens* R042 (Figure 4; Tag 43 in the floccular sludge, which is equivalent to Tag 17 in the granular sludge) was highly abundant in the floccular sludge and was decreased substantially during granulation.^19^ Although it is not yet possible to determine whether the community shift from the potential QQ organisms to QS organisms is a direct cause of granulation, or whether granulation favours the enrichment of QS organisms over the QQ species, the strong associations between both QS-dominated communities and granules, and QQ-dominated communities and flocs, suggest that differential AHL signalling is important in community level functions, such as granulation.

The co-occurrence of QS and QQ activities identified here is likely to be common across many habitats. Indeed, organisms known to be AHL producers or quenchers, including Proteobacteria, Bacteriodetes, Firmicutes and Actinobacteria phyla, have been recently expanded to also include Cyanobacteria,^44,45^ Acidobacteria,^46^ Bacteriodetes^47–49^ and Archaea^50^ from a diverse range of environments. Further, many of these species co-exist in the same niches, such as the rumen,^15,51^ microbial mats^14^ and the soil/rhizosphere.^7,8^ This is also likely the case for used water treatment facilities, which harbour very high microbial diversity, including many QS and QQ organisms as identified in this study and in the literature.^6,17^ Indeed, our preliminary metagenomic analysis and expression studies suggest that multiple QS and QQ genes are present in the activated sludge community from a full-scale plant (data not shown), not only supporting the co-occurrence of QS and QQ activities in the bioreactor community but also that community behaviour is controlled by the combined activity of the QS and QQ organisms present, as well as the specificity of the QQ activity for particular AHL signals.

In conclusion, it is clear from this study that while a positive correlation between AHL signalling and the assembly of microbial granular biofilms has been previously established,^19^ the detailed mechanisms by which community members with distinct QS and QQ activities interact to regulate QS behaviours at the community level remain to be defined. This study has developed the granulation system as an experimentally tractable system comprising high species diversity to investigate and determine, in a mechanistic fashion, how such communities control complex system level behaviours, such as granulation. Our study provides strong evidence that AHL-mediated QS signalling is a true community trait, with >65% of community members of diverse taxa engaged in the signalling and quenching processes. The dynamic abundance of QS and QQ organisms, as well as their respective signalling and quenching kinetics, appear to have a key role for defining the overall signal gradient, and hence regulate the transition from floccular to granular biofilms. Importantly, these findings may also imply that complex community assembly is a developmental process driven by QS signalling, with the enrichment of specific community members with QS signalling properties and exclusion of members with anti-QS signalling traits. The observation that QS and QQ activities are associated with phylogenetically diverse microbial species that are commonly found in many different natural habitats, in engineered ecosystems as well as in clinical settings, strongly suggests that AHL-mediated community communication could be a general feature of complex communities, and that the QS and QQ activities may have a significant role in the selection of stable and functional microbial assemblages.
