# Supplementary material for: Community quorum sensing signalling and quenching: microbial granular biofilm assembly
Source: NPJ Biofilms Microbiomes. 2015 May 27;1:15006–. doi: 10.1038/npjbiofilms.2015.6 (PMC5515215; doi:10.1038/npjbiofilms.2015.6)
Supplement: Supplementary Table S1 [file npjbiofilms20156-s1.doc]

**Table S1.** Bacterial strains used in this study.

| **Strain** | **Genotype and/or phenotype^1^** | **Reference or source** |
| --- | --- | --- |
| *A. tumefaciens* A136 | AHL biosensor, carrying pCF218 plasmid with *traR*, and pCF372 plasmid with P*_traI_-lacZ*; Tc^R^, Sp^R^ | [^1^](#_ENREF_1) |
| *C. violaceum* CV026 | AHL biosensor a double mini-Tn5 mutant from *Chrobacterium violaceum* ATCC 31532; Kan^R^ | [^2^](#_ENREF_2) |
| *E. coli* JM109 | *rec A1 endA1 gyrA96 thi hsdR17 supE44 relA1* Δ *(lac-proAB) mcrA* [*F’ traD36 proAB lacIq lacZ* ΔM15] | [^3^](#_ENREF_3) |
| *E. coli* JBA357 | AHL biosensor, carrying pJBA89 plasmid with *luxR-P_luxI_*-RBSII-*gfp*; Amp^R^ | [^4^](#_ENREF_4) |
| *P. aeruginosa* MH602 | AHL-producing strain, carrying a pMH602-*gfp* plasmid; Gm^R^ | [^5^](#_ENREF_5) |

^1^Abbreviations : Tc^R^, tetracycline resistance; Sp^R^, spectinomycin resistance; Kan^R^, kanamycin resistance; Amp^R^, ampicillin resistance; Gm^R^, gentamycin resistance; *gfp*, green fluorescent protein.

**Supplementary References**

1 Fuqua, C, Burbea, M & Winans, SC. Activity of the *Agrobacterium* Ti plasmid conjugal transfer regulator TraR is inhibited by the product of the *traM* gene. *J Bacteriol* 1995; **177**: 1367-1373.

2 McClean, KH, Winson, MK, Fish, L, Taylor, A, Chhabra, SR, Camara, M *et al.* Quorum sensing and *Chromobacterium violaceum*: exploitation of violacein production and inhibition for the detection of *N*-acylhomoserine lactones. *Microbiology* 1997; **143**: 3703-3711.

3 Yanisch-Perron, C, Vieira, J & Messing, J. Improved M13 phage cloning vectors and host strains: nucleotide sequences of the M13mp18 and pUC19 vectors. *Gene* 1985; **33**: 103-119.

4 Andersen, JB, Heydorn, A, Hentzer, M, Eberl, L, Geisenberger, O, Christensen, BB *et al.* *gfp*-based *N*-acyl homoserine-lactone sensor systems for detection of bacterial communication. *Appl Environ Microbiol* 2001; **67**: 575-585.

5 Heydorn, A, Ersbøll, B, Kato, J, Hentzer, M, Parsek, MR, Tolker-Nielsen, T *et al.* Statistical analysis of *Pseudomonas aeruginosa* biofilm development: impact of mutations in genes involved in twitching motility, cell-to-cell signaling, and stationary-phase sigma factor expression. *Appl Environ Microbiol* 2002; **68**: 2008-2017.
