# Supplementary material for: Community quorum sensing signalling and quenching: microbial granular biofilm assembly
Source: NPJ Biofilms Microbiomes. 2015 May 27;1:15006–. doi: 10.1038/npjbiofilms.2015.6 (PMC5515215; doi:10.1038/npjbiofilms.2015.6)
Supplement: Supplementary Table S2 [file npjbiofilms20156-s2.doc]

**Table S2.** Quorum sensing and quorum quenching profiles of individual isolates.

| **Strain Information** | | **Closest Match** | | | **QS^1^** | **QQ^2^** | | |
| --- | --- | --- | --- | --- | --- | --- | --- | --- |
| **Strain ID** | **NCBI Accession No.** | **NCBI ID** | **NCBI Accession No.** | **% of Matching** | **Bio_MS^3^** | **3OC6-HSL** | **3OC8-HSL** | **3OC12-HSL** |
| **Alpha-proteobacteria** | | | | | | | | |
| A009 | KC252645 | *Agrobacterium tumefaciens* strain 137 | FR828338.1 | 100 | -- | -- | -- | -- |
| A010 | KC252646 | *Agrobacterium tumefaciens* strain 137 | FR828338.1 | 100 | -- | -- | -- | -- |
| A011 | KC252647 | *Agrobacterium tumefaciens* strain 137 | FR828338.1 | 100 | -- | -- | -- | -- |
| A012 | KC252648 | *Agrobacterium tumefaciens* strain 137 | FR828338.1 | 100 | -- | -- | -- | -- |
| A026N | KC252662 | *Agrobacterium tumefaciens* strain 137 | FR828338.1 | 100 | -- | -- | -- | -- |
| N027b | KC252701 | *Agrobacterium tumefaciens* strain 137 | FR828338.1 | 100 | -- | -- | -- | -- |
| R007 | KC252836 | *Agrobacterium tumefaciens* strain 137 | FR828338.1 | 100 | -- | -- | -- | -- |
| R008 | KC252837 | *Agrobacterium tumefaciens* strain 137 | FR828338.1 | 100 | -- | -- | -- | -- |
| R017N | KC252848 | *Agrobacterium tumefaciens* strain 137 | FR828338.1 | 100 | -- | -- | -- | -- |
| R093N | KC252927 | *Bosea* sp. Dv3 | FJ774000.1 | 99 | -- | -- | + | + |
| R096 | KC252930 | *Bosea* sp. Dv3 | FJ774000.1 | 99 | -- | + | + | + |
| N008 | KC252681 | *Bosea thiooxidans* strain Q8 | FJ581444.1 | 99 | -- | + | +++ | +++ |
| N014 | KC252686 | *Bosea thiooxidans* strain Q8 | FJ581444.1 | 99 | -- | -- | -- | +++ |
| N026 | KC252699 | *Bosea thiooxidans* strain Q8 | FJ581444.1 | 99 | -- | + | + | +++ |
| N001 | KC252673 | *Bosea vestrisii* strain 34635 | NR_028799.1 | 99 | -- | + | + | +++ |
| N031 | KC252705 | *Bosea vestrisii* strain 34635 | NR_028799.1 | 99 | -- | + | + | +++ |
| R045 | KC252874 | *Bosea vestrisii* strain 34635 | NR_028799.1 | 99 | -- | + | + | +++ |
| R071N | KC252905 | *Bosea vestrisii* strain 34635 | NR_028799.1 | 99 | -- | + | + | + |
| R078N | KC252912 | *Bosea vestrisii* strain 34635 | NR_028799.1 | 99 | -- | +++ | +++ | + |
| N018 | KC252690 | *Bosea vestrisii* strain 34635 | NR_028799.1 | 99 | -- | -- | -- | -- |
| N061 | KC252734 | *Brevundimonas diminuta* strain 3P04AD | EU977701.1 | 100 | -- | + | +++ | +++ |
| P025 | KC252762 | *Brevundimonas diminuta* strain 3P04AD | EU977701.1 | 100 | -- | +++ | +++ | +++ |
| P051 | KC252792 | *Brevundimonas diminuta* strain 3P04AD | EU977701.1 | 100 | -- | +++ | +++ | +++ |
| P053 | KC252794 | *Brevundimonas diminuta* strain 3P04AD | EU977701.1 | 100 | -- | +++ | +++ | +++ |
| R057 | KC252887 | *Brevundimonas diminuta* strain 3P04AD | EU977701.1 | 100 | -- | +++ | +++ | +++ |
| R043 | KC252872 | *Brevundimonas olei* strain MJ15 | GQ250440.2 | 98 | -- | -- | -- | + |

| **Strain Information** | | **Closest Match** | | | **QS^1^** | | **QQ^2^** | | | | |
| --- | --- | --- | --- | --- | --- | --- | --- | --- | --- | --- | --- |
| **Strain ID** | **NCBI Accession**  **No.** | **NCBI ID** | **NCBI Accession No.** | **% of Matching** | **Bio_MS^3^** | | **3OC6-**  **HSL** | | **3OC8-**  **HSL** | | **3OC12-**  **HSL** |
| **Alpha-proteobacteria** | | | | | | | | | | | |
| R095 | KC252929 | *Mesorhizobium ciceri* strain NBRC 100389 | AB681164.1 | 98 | -- | | -- | | -- | | +++ |
| N002N | KC252674 | *Novosphingobium* sp. FW-6 | JQ349046.1 | 98 | -- | | + | | +++ | | +++ |
| N058 | KC252730 | *Ochrobactrum anthropi* strain W-7 | EU187487.1 | 100 | -- | | +++ | | +++ | | +++ |
| R005a | KC252833 | *Ochrobactrum anthropi* strain W-7 | EU187487.1 | 100 | -- | | +++ | | +++ | | +++ |
| R006 | KC252835 | *Ochrobactrum anthropi* strain W-7 | EU187487.1 | 100 | -- | | +++ | | +++ | | +++ |
| R058 | KC252888 | *Ochrobactrum anthropi* strain W-7 | EU187487.1 | 100 | -- | | +++ | | +++ | | +++ |
| N065 | KC252738 | *Rhizobium borbori* strain DN365 | GU356639.1 | 99 | +++ | | +++ | | +++ | | +++ |
| R055 | KC252885 | *Rhizobium borbori* strain DN365 | GU356639.1 | 98 | +++ | | -- | | -- | | -- |
| R087 | KC252921 | *Rhodobacter gluconicum* | AB077986.1 | 99 | -- | | -- | | -- | | -- |
| R023 | KC252851 | *Rhodobacter maris* strain JA276 | NR_042629.1 | 97 | +++ | | -- | | -- | | -- |
| R074 | KC252908 | *Rhodobacter maris* strain JA276 | NR_042629.1 | 97 | +++ | | -- | | -- | | -- |
| R076N | KC252910 | *Rhodobacter maris* strain JA276 | NR_042629.1 | 97 | +++ | | -- | | -- | | -- |
| S009a | KC252938 | *Rhodobacter maris* strain JA276 | NR_042629.1 | 97 | +++ | | -- | | -- | | -- |
| S011a | KC252941 | *Rhodobacter maris* strain JA276 | NR_042629.1 | 97 | +++ | | -- | | -- | | -- |
| N020 | KC252692 | *Rhodobacter* sp. XJ-1 | GU184187.1 | 99 | -- | | -- | | -- | | -- |
| R077N | KC252911 | *Rhodobacter* sp. XJ-1 | GU184187.1 | 100 | -- | | -- | | -- | | +++ |
| R049 | KC252878 | *Roseomonas terrae* strain DS-48 | NR_044188.1 | 99 | -- | | +++ | | +++ | | +++ |
| R089 | KC252923 | *Sphingobium yanoikuyae* strain R5-725 | JQ659783.1 | 99 | -- | | -- | | -- | | -- |
| R054a | KC252883 | *Sphingomonas* sp. PA225 | AM900788.1 | 96 | +++ | | -- | | -- | | -- |
| R060 | KC252890 | *Sphingomonas* sp. PA225 | AM900788.1 | 96 | +++ | | -- | | -- | | + |
| N016 | KC252688 | *Shinella fusca* strain DC-196T | FM177879.1 | 100 | +++ | | -- | | -- | | -- |
| **Beta-proteobacteria** | | | | | | | | | | | |
| P070N | KC252810 | *Achromobacter spanius* strain G191 | JN629044.1 | 99 | -- | -- | | -- | | -- | |
| A013 | KC252649 | *Achromobacter xylosoxidans* strain NCPP-44 | AB547225.1 | 100 | -- | -- | | -- | | -- | |
| A014 | KC252650 | *Achromobacter xylosoxidans* strain NCPP-44 | AB547225.1 | 100 | -- | -- | | -- | | -- | |
| A015 | KC252651 | *Achromobacter xylosoxidans* strain NCPP-44 | AB547225.1 | 100 | -- | -- | | -- | | -- | |

**Table S2.** Continued.

**Table S2.** Continued.

| **Strain Information** | | **Closest Match** | | | **QS^1^** | **QQ^2^** | | |
| --- | --- | --- | --- | --- | --- | --- | --- | --- |
| **Strain ID** | **NCBI Accession**  **No.** | **NCBI ID** | **NCBI Accession No.** | **% of Matching** | **Bio_MS^3^** | **3OC6-HSL** | **3OC8-HSL** | **3OC12-HSL** |
| **Beta-proteobacteria** | | | | | | | | |
| A016N | KC252652 | *Achromobacter xylosoxidans* strain NCPP-44 | AB547225.1 | 100 | -- | -- | -- | -- |
| A017 | KC252653 | *Achromobacter xylosoxidans* strain NCPP-44 | AB547225.1 | 100 | -- | -- | -- | -- |
| A023 | KC252659 | *Achromobacter xylosoxidans* strain NCPP-44 | AB547225.1 | 100 | -- | -- | -- | -- |
| A029 | KC252664 | *Achromobacter xylosoxidans* strain NCPP-44 | AB547225.1 | 100 | -- | -- | -- | -- |
| A030 | KC252665 | *Achromobacter xylosoxidans* strain NCPP-44 | AB547225.1 | 100 | -- | -- | -- | -- |
| A031 | KC252666 | *Achromobacter xylosoxidans* strain NCPP-44 | AB547225.1 | 100 | -- | -- | -- | -- |
| A032 | KC252667 | *Achromobacter xylosoxidans* strain NCPP-44 | AB547225.1 | 100 | -- | -- | -- | -- |
| A033 | KC252668 | *Achromobacter xylosoxidans* strain NCPP-44 | AB547225.1 | 100 | -- | -- | -- | -- |
| A034 | KC252669 | *Achromobacter xylosoxidans* strain NCPP-44 | AB547225.1 | 100 | -- | -- | -- | -- |
| A035 | KC252670 | *Achromobacter xylosoxidans* strain NCPP-44 | AB547225.1 | 100 | -- | -- | -- | -- |
| A036 | KC252671 | *Achromobacter xylosoxidans* strain NCPP-44 | AB547225.1 | 100 | -- | -- | -- | -- |
| P029 | KC252767 | *Achromobacter xylosoxidans* strain NCPP-44 | AB547225.1 | 100 | -- | -- | -- | -- |
| P072 | KC252812 | *Achromobacter xylosoxidans* strain NCPP-44 | AB547225.1 | 100 | -- | -- | -- | -- |
| N035b | KC252710 | *Acidovorax delafieldii* strain PCWCS4 | GQ284437.1 | 100 | -- | -- | -- | +++ |
| R081 | KC252915 | *Acidovorax facilis* strain 228 | EU730927.1 | 100 | +++ | -- | -- | -- |
| P062 | KC252803 | *Alcaligenes* sp. LSBA1977 | FJ908708.1 | 99 | -- | -- | -- | -- |
| R042* | KC252871 | *Diaphorobacter nitroreducens* strain NA10B | NR_024782.1 | 96 | -- | -- | -- | +++ |
| P027 | KC252765 | *Delftia tsuruhatensis* strain BN-HKY6 | HQ731453.1 | 100 | -- | -- | +++ | +++ |
| P035a | KC252770 | *Delftia tsuruhatensis* strain BN-HKY6 | HQ731453.1 | 100 | -- | -- | +++ | +++ |
| P039 | KC252776 | *Delftia tsuruhatensis* strain BN-HKY6 | HQ731453.1 | 100 | -- | -- | +++ | +++ |
| R025N | KC252853 | *Hydrogenophaga flava* strain NBRC 102514 | AB681848.1 | 99 | -- | -- | -- | -- |
| R066 | KC252896 | *Paludibacterium yongneupense* strain 5YN8-15 | NR_042552.1 | 97 | -- | -- | -- | -- |
| R052 | KC252881 | *Thauera selenatis* strain AX39 | NR_026474.1 | 99 | -- | -- | -- | -- |
| R086 | KC252920 | *Thauera selenatis* strain AX39 | NR_026474.1 | 99 | -- | -- | -- | -- |
| S004b | KC252935 | *Variovorax paradoxus* strain E4C | AF209469.1 | 100 | -- | -- | + | +++ |

| **Strain Information** | | **Closest Match** | | | **QS^1^** | **QQ^2^** | | |
| --- | --- | --- | --- | --- | --- | --- | --- | --- |
| **Strain ID** | **NCBI Accession**  **No.** | **NCBI ID** | **NCBI Accession No.** | **% of Matching** | **Bio_MS^3^** | **3OC6-HSL** | **3OC8-HSL** | **3OC12-HSL** |
| **Gamma-proteobacteria** | | | | | | | | |
| N024 | KC252697 | *Acinetobacter junii* strain tu13 | FJ544395.1 | 99 | -- | -- | -- | +++ |
| N029N | KC252703 | *Acinetobacter junii* strain tu13 | FJ544395.1 | 99 | -- | -- | -- | +++ |
| N030N | KC252704 | *Acinetobacter junii* strain tu13 | FJ544395.1 | 99 | -- | -- | -- | +++ |
| S003 | KC252934 | *Acinetobacter junii* strain tu13 | FJ544395.1 | 99 | -- | -- | -- | +++ |
| S010 | KC252940 | *Acinetobacter junii* strain tu13 | FJ544395.1 | 99 | -- | -- | -- | +++ |
| R088 | KC252922 | *Acinetobacter schindleri* strain W1-2 | FJ373024.1 | 99 | -- | -- | -- | -- |
| R027N | KC252855 | *Acinetobacter* sp. 8A18N1 | HQ246229.1 | 100 | -- | -- | -- | +++ |
| R028 | KC252856 | *Acinetobacter* sp. 8A18N1 | HQ246229.1 | 100 | -- | -- | -- | +++ |
| S005 | KC252936 | *Acinetobacter* sp. 8A18N1 | HQ246229.1 | 100 | -- | -- | -- | +++ |
| P054 | KC252795 | *Aeromonas caviae* strain JXZ-3 | JF496552.1 | 100 | -- | -- | -- | -- |
| P055 | KC252796 | *Aeromonas caviae* strain JXZ-3 | JF496552.1 | 100 | -- | -- | -- | -- |
| P086 | KC252824 | *Aeromonas caviae* strain JXZ-3 | JF496552.1 | 100 | -- | -- | -- | -- |
| P087 | KC252825 | *Aeromonas caviae* strain JXZ-3 | JF496552.1 | 100 | -- | -- | -- | -- |
| P005 | KC252743 | *Citrobacter freundii* strain MRB070408-2 | GU126683.1 | 99 | -- | -- | -- | -- |
| P006 | KC252744 | *Citrobacter freundii* strain MRB070408-2 | GU126683.1 | 99 | -- | -- | -- | -- |
| P009 | KC252747 | *Citrobacter freundii* strain MRB070408-2 | GU126683.1 | 99 | -- | -- | -- | -- |
| P010N | KC252748 | *Citrobacter freundii* strain MRB070408-2 | GU126683.1 | 99 | -- | -- | -- | -- |
| P013N | KC252751 | *Citrobacter freundii* strain MRB070408-2 | GU126683.1 | 99 | -- | -- | -- | -- |
| P014 | KC252752 | *Citrobacter freundii* strain MRB070408-2 | GU126683.1 | 99 | -- | -- | -- | -- |
| P016N | KC252754 | *Citrobacter freundii* strain MRB070408-2 | GU126683.1 | 99 | -- | -- | -- | -- |
| P019 | KC252756 | *Citrobacter freundii* strain MRB070408-2 | GU126683.1 | 99 | -- | -- | -- | -- |
| P020 | KC252757 | *Citrobacter freundii* strain MRB070408-2 | GU126683.1 | 99 | -- | -- | -- | -- |
| P023 | KC252760 | *Citrobacter freundii* strain MRB070408-2 | GU126683.1 | 99 | -- | -- | -- | -- |
| P024 | KC252761 | *Citrobacter freundii* strain MRB070408-2 | GU126683.1 | 99 | -- | -- | -- | -- |
| P028N | KC252766 | *Citrobacter freundii* strain MRB070408-2 | GU126683.1 | 99 | -- | -- | -- | -- |
| P035b | KC252771 | *Citrobacter freundii* strain MRB070408-2 | GU126683.1 | 99 | -- | -- | -- | -- |

**Table S2.** Continued.

**Table S2.** Continued.

| **Strain Information** | | **Closest Match** | | | **QS^1^** | **QQ^2^** | | |
| --- | --- | --- | --- | --- | --- | --- | --- | --- |
| **Strain ID** | **NCBI Accession**  **No.** | **NCBI ID** | **NCBI Accession No.** | **% of Matching** | **Bio_MS^3^** | **3OC6-HSL** | **3OC8-HSL** | **3OC12-HSL** |
| **Gamma-proteobacteria** | | | | | | | | |
| P036 | KC252772 | *Citrobacter freundii* strain MRB070408-2 | GU126683.1 | 99 | -- | -- | -- | -- |
| P037 | KC252773 | *Citrobacter freundii* strain MRB070408-2 | GU126683.1 | 99 | -- | -- | -- | -- |
| P038b | KC252775 | *Citrobacter freundii* strain MRB070408-2 | GU126683.1 | 99 | -- | -- | -- | -- |
| P041a | KC252778 | *Citrobacter freundii* strain MRB070408-2 | GU126683.1 | 99 | -- | -- | -- | -- |
| P042bN | KC252782 | *Citrobacter freundii* strain MRB070408-2 | GU126683.1 | 99 | -- | -- | -- | -- |
| P043N | KC252783 | *Citrobacter freundii* strain MRB070408-2 | GU126683.1 | 99 | -- | -- | -- | -- |
| P046a | KC252786 | *Citrobacter freundii* strain MRB070408-2 | GU126683.1 | 99 | -- | -- | -- | -- |
| P061 | KC252802 | *Citrobacter freundii* strain MRB070408-2 | GU126683.1 | 99 | -- | -- | -- | -- |
| P065 | KC252806 | *Citrobacter freundii* strain MRB070408-2 | GU126683.1 | 99 | -- | -- | -- | -- |
| P073 | KC252813 | *Citrobacter freundii* strain MRB070408-2 | GU126683.1 | 99 | -- | -- | -- | -- |
| P074 | KC252814 | *Citrobacter freundii* strain MRB070408-2 | GU126683.1 | 99 | -- | -- | -- | -- |
| N063 | KC252736 | *Enterobacter ludwigii* strain BIHB 336 | FJ859683.1 | 99 | -- | -- | -- | -- |
| N027a | KC252700 | *Frateuria* sp. Ni-H2-1 | EU170476.1 | 100 | +++ | -- | -- | + |
| R011a | KC252840 | *Frateuria* sp. Ni-H2-1 | EU170476.1 | 100 | +++ | -- | -- | + |
| R037 | KC252866 | *Lysobacter brunescens* strain F2 | GQ859167.1 | 100 | +++ | -- | -- | +++ |
| R044 | KC252873 | *Lysobacter brunescens* strain F2 | GQ859167.1 | 100 | +++ | -- | -- | +++ |
| R053 | KC252882 | *Lysobacter brunescens* strain F2 | GQ859167.1 | 100 | +++ | -- | -- | +++ |
| R068a | KC252901 | *Lysobacter brunescens* strain F2 | GQ859167.1 | 100 | +++ | -- | -- | +++ |
| R069 | KC252903 | *Lysobacter brunescens* strain F2 | GQ859167.1 | 100 | +++ | -- | -- | +++ |
| R072 | KC252906 | *Lysobacter brunescens* strain F2 | GQ859167.1 | 100 | +++ | -- | -- | +++ |
| R082 | KC252916 | *Lysobacter brunescens* strain F2 | GQ859167.1 | 100 | +++ | -- | -- | +++ |
| R092 | KC252926 | *Lysobacter brunescens* strain F2 | GQ859167.1 | 100 | +++ | -- | -- | +++ |
| R094 | KC252928 | *Lysobacter brunescens* strain F2 | GQ859167.1 | 100 | +++ | -- | -- | +++ |
| R097 | KC252931 | *Lysobacter brunescens* strain F2 | GQ859167.1 | 100 | +++ | -- | -- | +++ |
| P058 | KC252799 | *Klebsiella pneumoniae* strain SDM45 | GU997596.1 | 99 | -- | -- | -- | -- |
| R067c | KC252899 | *Pantoea ananatis* strain C-2-3 | GU324769.1 | 99 | -- | -- | -- | -- |

| **Strain Information** | | **Closest Match** | | | **QS^1^** | **QQ^2^** | | |
| --- | --- | --- | --- | --- | --- | --- | --- | --- |
| **Strain ID** | **NCBI Accession**  **No.** | **NCBI ID** | **NCBI Accession No.** | **% of Matching** | **Bio_MS^3^** | **3OC6-HSL** | **3OC8-HSL** | **3OC12-HSL** |
| **Gamma-proteobacteria** | | | | | | | | |
| R067d | KC252900 | *Pantoea stewartii subsp. stewartii* strain GSPB 2626 | AF373198.1 | 99 | +++ | -- | -- | -- |
| P001 | KC252739 | *Pseudomonas otitidis* strain TNAU 45 | GQ339108.1 | 99 | -- | -- | -- | +++ |
| P038a | KC252774 | *Pseudomonas otitidis* strain TNAU 45 | GQ339108.1 | 99 | -- | -- | -- | +++ |
| P040 | KC252777 | *Pseudomonas otitidis* strain TNAU 45 | GQ339108.1 | 99 | -- | -- | -- | +++ |
| P046b | KC252787 | *Pseudomonas otitidis* strain TNAU 45 | GQ339108.1 | 99 | -- | -- | -- | +++ |
| P085 | KC252823 | *Pseudomonas otitidis* strain TNAU 45 | GQ339108.1 | 99 | -- | -- | -- | +++ |
| P090 | KC252828 | *Pseudomonas otitidis* strain TNAU 45 | GQ339108.1 | 99 | -- | -- | -- | +++ |
| P080 | KC252818 | *Pseudomonas veronii* strain ZW | GU357489.1 | 99 | -- | -- | -- | + |
| P060 | KC252801 | *Pseudoxanthomonas japonensis* strain NBRC 101033 | AB681337.1 | 100 | -- | -- | -- | +++ |
| P079N | KC252817 | *Pseudoxanthomonas japonensis* strain NBRC 101033 | AB681337.1 | 100 | -- | -- | -- | +++ |
| N012 | KC252684 | *Pseudoxanthomonas* sp. 11_4K | EF540482.1 | 99 | -- | -- | -- | +++ |
| R083 | KC252917 | *Rheinheimera chironomi* strain EIF90 | HM480359.1 | 99 | -- | -- | -- | +++ |
| A005 | KC252640 | *Stenotrophomonas maltophilia* strain CCF0025 | GU391033.1 | 100 | -- | -- | -- | + |
| A006 | KC252641 | *Stenotrophomonas maltophilia* strain CCF0025 | GU391033.1 | 100 | -- | -- | -- | + |
| A007 | KC252642 | *Stenotrophomonas maltophilia* strain CCF0025 | GU391033.1 | 100 | -- | -- | -- | + |
| A008a | KC252643 | *Stenotrophomonas maltophilia* strain CCF0025 | GU391033.1 | 100 | -- | -- | -- | -- |
| A019 | KC252655 | *Stenotrophomonas maltophilia* strain CCF0025 | GU391033.1 | 100 | -- | -- | -- | + |
| A021N | KC252657 | *Stenotrophomonas maltophilia* strain CCF0025 | GU391033.1 | 100 | -- | -- | -- | -- |
| A022 | KC252658 | *Stenotrophomonas maltophilia* strain CCF0025 | GU391033.1 | 100 | -- | -- | -- | + |
| A027 | KC252663 | *Stenotrophomonas maltophilia* strain CCF0025 | GU391033.1 | 100 | -- | -- | -- | + |
| N005 | KC252677 | *Stenotrophomonas maltophilia* strain CCF0025 | GU391033.1 | 100 | -- | -- | -- | + |
| P003N | KC252741 | *Stenotrophomonas maltophilia* strain CCF0025 | GU391033.1 | 100 | -- | -- | -- | -- |
| P007 | KC252745 | *Stenotrophomonas maltophilia* strain CCF0025 | GU391033.1 | 100 | -- | -- | -- | -- |

**Table S2.** Continued.

**Table S2.** Continued.

| **Strain Information** | | **Closest Match** | | | **QS^1^** | **QQ^2^** | | |
| --- | --- | --- | --- | --- | --- | --- | --- | --- |
| **Strain ID** | **NCBI Accession**  **No.** | **NCBI ID** | **NCBI Accession No.** | **% of Matching** | **Bio_MS^3^** | **3OC6-HSL** | **3OC8-HSL** | **3OC12-HSL** |
| **Gamma-proteobacteria** | | | | | | | | |
| P008 | KC252746 | *Stenotrophomonas maltophilia* strain CCF0025 | GU391033.1 | 100 | -- | -- | -- | -- |
| P011 | KC252749 | *Stenotrophomonas maltophilia* strain CCF0025 | GU391033.1 | 100 | -- | -- | -- | -- |
| P012 | KC252750 | *Stenotrophomonas maltophilia* strain CCF0025 | GU391033.1 | 100 | -- | -- | -- | -- |
| P015 | KC252753 | *Stenotrophomonas maltophilia* strain CCF0025 | GU391033.1 | 100 | -- | -- | -- | -- |
| P017N | KC252755 | *Stenotrophomonas maltophilia* strain CCF0025 | GU391033.1 | 100 | -- | -- | -- | + |
| P021 | KC252758 | *Stenotrophomonas maltophilia* strain CCF0025 | GU391033.1 | 100 | -- | -- | -- | + |
| P022 | KC252759 | *Stenotrophomonas maltophilia* strain CCF0025 | GU391033.1 | 100 | -- | -- | -- | + |
| P056 | KC252797 | *Stenotrophomonas maltophilia* strain CCF0025 | GU391033.1 | 100 | -- | -- | -- | -- |
| P059 | KC252800 | *Stenotrophomonas maltophilia* strain CCF0025 | GU391033.1 | 100 | -- | -- | -- | + |
| P064N | KC252805 | *Stenotrophomonas maltophilia* strain CCF0025 | GU391033.1 | 100 | -- | -- | -- | -- |
| P067 | KC252808 | *Stenotrophomonas maltophilia* strain CCF0025 | GU391033.1 | 100 | -- | -- | -- | -- |
| P069 | KC252809 | *Stenotrophomonas maltophilia* strain CCF0025 | GU391033.1 | 100 | -- | -- | -- | -- |
| P071 | KC252811 | *Stenotrophomonas maltophilia* strain CCF0025 | GU391033.1 | 100 | -- | -- | -- | -- |
| P075 | KC252815 | *Stenotrophomonas maltophilia* strain CCF0025 | GU391033.1 | 100 | -- | -- | -- | + |
| P076 | KC252816 | *Stenotrophomonas maltophilia* strain CCF0025 | GU391033.1 | 100 | -- | -- | -- | + |
| N017 | KC252689 | *Stenotrophomonas maltophilia* strain SPd | FJ405363.1 | 99 | -- | -- | -- | + |
| N022b | KC252696 | *Stenotrophomonas maltophilia* strain SPd | FJ405363.1 | 99 | -- | -- | -- | -- |
| N036 | KC252712 | *Stenotrophomonas maltophilia* strain YSP48 | JF894170.1 | 99 | -- | -- | -- | + |
| R015N | KC252845 | *Stenotrophomonas maltophilia* strain YSP48 | JF894170.1 | 99 | -- | -- | -- | + |
| R040N | KC252869 | *Stenotrophomonas maltophilia* strain YSP48 | JF894170.1 | 99 | -- | -- | -- | + |
| N006NR | KC252678 | *Stenotrophomonas* sp. M2T2B9 | GQ246699.1 | 100 | -- | -- | -- | -- |
| N006NS | KC252679 | *Stenotrophomonas* sp. M2T2B9 | GQ246699.1 | 100 | -- | -- | -- | -- |
| N025 | KC252698 | *Stenotrophomonas* sp. M2T2B9 | GQ246699.1 | 100 | +++ | -- | -- | -- |
| N054 | KC252726 | *Stenotrophomonas* sp. M2T2B9 | GQ246699.1 | 100 | -- | -- | -- | -- |
| N059a | KC252731 | *Stenotrophomonas* sp. M2T2B9 | GQ246699.1 | 100 | +++ | -- | -- | -- |
| N060 | KC252733 | *Stenotrophomonas* sp. M2T2B9 | GQ246699.1 | 100 | -- | -- | -- | -- |

**Table S2.** Continued.

| **Strain Information** | | **Closest Match** | | | **QS^1^** | **QQ^2^** | | |
| --- | --- | --- | --- | --- | --- | --- | --- | --- |
| **Strain ID** | **NCBI Accession**  **No.** | **NCBI ID** | **NCBI Accession No.** | **% of Matching** | **Bio_MS^3^** | **3OC6-HSL** | **3OC8-HSL** | **3OC12-HSL** |
| **Gamma-proteobacteria** | | | | | | | | |
| N064 | KC252737 | *Stenotrophomonas* sp. M2T2B9 | GQ246699.1 | 100 | +++ | -- | -- | -- |
| P002 | KC252740 | *Stenotrophomonas* sp. M2T2B9 | GQ246699.1 | 100 | +++ | -- | -- | -- |
| P026NR | KC252763 | *Stenotrophomonas* sp. M2T2B9 | GQ246699.1 | 100 | +++ | -- | -- | -- |
| P026NS | KC252764 | *Stenotrophomonas* sp. M2T2B9 | GQ246699.1 | 100 | +++ | -- | -- | +++ |
| P042aR | KC252780 | *Stenotrophomonas* sp. M2T2B9 | GQ246699.1 | 100 | -- | -- | -- | -- |
| P042aS | KC252781 | *Stenotrophomonas* sp. M2T2B9 | GQ246699.1 | 100 | -- | -- | -- | -- |
| P045 | KC252785 | *Stenotrophomonas* sp. M2T2B9 | GQ246699.1 | 100 | +++ | -- | -- | -- |
| P063 | KC252804 | *Stenotrophomonas* sp. M2T2B9 | GQ246699.1 | 100 | +++ | -- | -- | -- |
| P088 | KC252826 | *Stenotrophomonas* sp. M2T2B9 | GQ246699.1 | 100 | +++ | -- | -- | -- |
| R063 | KC252893 | *Stenotrophomonas* sp. M2T2B9 | GQ246699.1 | 100 | +++ | -- | -- | + |
| R038 | KC252867 | *Thermomonas brevis* strain R-13291 | NR_025578.1 | 97 | -- | -- | -- | -- |
| R039N | KC252868 | *Thermomonas brevis* strain R-13291 | NR_025578.1 | 97 | -- | -- | -- | -- |
| R051* | KC252880 | *Dokdonella* sp*.* CC-YHH031 | GQ281768.1 | 94 | -- | -- | -- | +++ |
| **Actinobacteria** | | | | | | | | |
| N038 | KC252714 | *Agromyces mediolanus* strain CNF186 | D45054.1 | 99 | -- | -- | -- | -- |
| N021b | KC252694 | *Brevibacterium aureum* strain Enb17 | AY299093.1 | 99 | -- | -- | -- | +++ |
| N035c | KC252711 | *Brevibacterium aureum* strain Enb17 | AY299093.1 | 99 | -- | -- | -- | +++ |
| N028 | KC252702 | *Microbacterium flavum* strain YM18-098 | NR_041562.1 | 100 | -- | + | -- | +++ |
| R024N | KC252852 | *Microbacterium flavum* strain YM18-098 | NR_041562.1 | 100 | -- | + | + | +++ |
| N056 | KC252728 | *Microbacterium hydrocarbonoxydans* strain HR98 | JF700471.1 | 98 | -- | -- | -- | + |
| R075N | KC252909 | *Microbacterium hydrocarbonoxydans* strain HR98 | JF700471.1 | 98 | -- | -- | -- | + |
| N019N | KC252691 | *Microbacterium laevaniformans* strain 1ck28 | JQ229794.1 | 100 | -- | -- | -- | + |
| N021a | KC252693 | *Microbacterium laevaniformans* strain 1ck28 | JQ229794.1 | 100 | -- | -- | -- | -- |
| N022a | KC252695 | *Microbacterium laevaniformans* strain 1ck28 | JQ229794.1 | 100 | -- | -- | -- | + |

**Table S2.** Continued.

| **Strain Information** | | **Closest Match** | | | **QS^1^** | **QQ^2^** | | |
| --- | --- | --- | --- | --- | --- | --- | --- | --- |
| **Strain ID** | **NCBI Accession**  **No.** | **NCBI ID** | **NCBI Accession No.** | **% of Matching** | **Bio_MS^3^** | **3OC6-HSL** | **3OC8-HSL** | **3OC12-HSL** |
| **Actinobacteria** | | | | | | | | |
| N039 | KC252715 | *Microbacterium laevaniformans* strain 1ck28 | JQ229794.1 | 100 | -- | -- | -- | + |
| N050 | KC252724 | *Microbacterium laevaniformans* strain 1ck28 | JQ229794.1 | 100 | -- | -- | -- | + |
| N050 | KC252724 | *Microbacterium laevaniformans* strain 1ck28 | JQ229794.1 | 100 | -- | -- | -- | + |
| N055 | KC252727 | *Microbacterium laevaniformans* strain 1ck28 | JQ229794.1 | 100 |  | -- | -- | + |
| R009 | KC252838 | *Microbacterium laevaniformans* strain 1ck28 | JQ229794.1 | 100 | -- | -- | -- | + |
| R010 | KC252839 | *Microbacterium laevaniformans* strain 1ck28 | JQ229794.1 | 100 | -- | -- | -- | + |
| R012N | KC252842 | *Microbacterium laevaniformans* strain 1ck28 | JQ229794.1 | 100 | -- | -- | -- | + |
| R013 | KC252843 | *Microbacterium laevaniformans* strain 1ck28 | JQ229794.1 | 100 | -- | -- | -- | + |
| R014N | KC252844 | *Microbacterium laevaniformans* strain 1ck28 | JQ229794.1 | 100 | -- | -- | -- | + |
| R026 | KC252854 | *Microbacterium laevaniformans* strain 1ck28 | JQ229794.1 | 100 | -- | -- | -- | + |
| R067a | KC252897 | *Microbacterium laevaniformans* strain 1ck28 | JQ229794.1 | 100 | -- | -- | -- | + |
| R070N | KC252904 | *Microbacterium laevaniformans* strain 1ck28 | JQ229794.1 | 100 | -- | -- | -- | + |
| R079 | KC252913 | *Microbacterium laevaniformans* strain 1ck28 | JQ229794.1 | 100 | -- | -- | -- | + |
| N037 | KC252713 | *Microbacterium oxydans* strain OL-4 | HQ202812.1 | 100 | -- | + | -- | +++ |
| N049 | KC252723 | *Microbacterium oxydans* strain OL-4 | HQ202812.1 | 100 | -- | + | -- | +++ |
| P032 | KC252769 | *Microbacterium oxydans* strain OL-4 | HQ202812.1 | 100 | -- | + | -- | +++ |
| P048a | KC252789 | *Microbacterium oxydans* strain OL-4 | HQ202812.1 | 100 | -- | + | -- | +++ |
| P052 | KC252793 | *Microbacterium oxydans* strain OL-4 | HQ202812.1 | 100 | -- | +++ | -- | +++ |
| P081 | KC252819 | *Microbacterium oxydans* strain OL-4 | HQ202812.1 | 100 | -- | + | -- | +++ |
| P082 | KC252820 | *Microbacterium oxydans* strain OL-4 | HQ202812.1 | 100 | -- | + | -- | +++ |
| P083 | KC252821 | *Microbacterium oxydans* strain OL-4 | HQ202812.1 | 100 | -- | + | -- | +++ |
| R073 | KC252907 | *Microbacterium oxydans* strain OL-4 | HQ202812.1 | 100 | -- | + | -- | +++ |
| R084 | KC252918 | *Microbacterium oxydans* strain OL-4 | HQ202812.1 | 100 | -- | + | -- | +++ |
| N041 | KC252716 | *Pimelobacter simplex* strain S151 | AY509240.1 | 99 | -- | -- | -- | +++ |
| N042 | KC252717 | *Pimelobacter simplex* strain S151 | AY509240.1 | 99 | -- | -- | -- | +++ |
| N044 | KC252718 | *Pimelobacter simplex* strain S151 | AY509240.1 | 99 | -- | -- | -- | +++ |

**Table S2.** Continued.

| **Strain Information** | | **Closest Match** | | | **QS^1^** | **QQ^2^** | | |
| --- | --- | --- | --- | --- | --- | --- | --- | --- |
| **Strain ID** | **NCBI Accession**  **No.** | **NCBI ID** | **NCBI Accession No.** | **% of Matching** | **Bio_MS^3^** | **3OC6-HSL** | **3OC8-HSL** | **3OC12-HSL** |
| **Actinobacteria** | | | | | | | | |
| N053 | KC252725 | *Pimelobacter simplex* strain S151 | AY509240.1 | 99 | -- | -- | -- | +++ |
| N057 | KC252729 | *Pimelobacter simplex* strain S151 | AY509240.1 | 99 | -- | -- | -- | +++ |
| N062 | KC252735 | *Pimelobacter simplex* strain S151 | AY509240.1 | 99 | -- | -- | -- | +++ |
| P047 | KC252788 | *Rhodococcus erythropolis* strain ZJB-0910 | GU726138.1 | 100 | -- | +++ | +++ | +++ |
| R011b | KC252841 | *Rhodococcus erythropolis* strain ZJB-0910 | GU726138.1 | 100 | -- | +++ | +++ | +++ |
| N003 | KC252675 | *Tsukamurella tyrosinosolvens* strain IFM 10623 | AB478956.1 | 100 | -- | -- | +++ | +++ |
| N034N | KC252708 | *Tsukamurella tyrosinosolvens* strain IFM 10623 | AB478956.1 | 100 | -- | -- | +++ | +++ |
| R019 | KC252849 | *Tsukamurella tyrosinosolvens* strain IFM 10623 | AB478956.1 | 100 | -- | -- | +++ | +++ |
| R020 | KC252850 | *Tsukamurella tyrosinosolvens* strain IFM 10623 | AB478956.1 | 100 | -- | -- | +++ | +++ |
| **Bacteriodetes** | | | | | | | | |
| A001 | KC252636 | *Chryseobacterium indologenes* strain McR-1 | JF894157.1 | 99 | -- | -- | -- | +++ |
| A002 | KC252637 | *Chryseobacterium indologenes* strain McR-1 | JF894157.1 | 99 | -- | -- | -- | + |
| A003 | KC252638 | *Chryseobacterium indologenes* strain McR-1 | JF894157.1 | 99 | -- | -- | -- | +++ |
| A004 | KC252639 | *Chryseobacterium indologenes* strain McR-1 | JF894157.1 | 99 | -- | -- | -- | +++ |
| A018 | KC252654 | *Chryseobacterium indologenes* strain McR-1 | JF894157.1 | 99 | -- | -- | -- | +++ |
| A020 | KC252656 | *Chryseobacterium indologenes* strain McR-1 | JF894157.1 | 99 | -- | -- | -- | +++ |
| A024 | KC252660 | *Chryseobacterium indologenes* strain McR-1 | JF894157.1 | 99 | -- | -- | -- | +++ |
| A025 | KC252661 | *Chryseobacterium indologenes* strain McR-1 | JF894157.1 | 99 | -- | -- | -- | +++ |
| A037 | KC252672 | *Chryseobacterium indologenes* strain McR-1 | JF894157.1 | 99 | -- | -- | -- | +++ |
| N004 | KC252676 | *Chryseobacterium indologenes* strain McR-1 | JF894157.1 | 99 | -- | -- | -- | +++ |
| N032 | KC252706 | *Chryseobacterium indologenes* strain McR-1 | JF894157.1 | 99 | -- | -- | -- | +++ |
| N033 | KC252707 | *Chryseobacterium indologenes* strain McR-1 | JF894157.1 | 99 | -- | -- | -- | +++ |

**Table S2.** Continued.

| **Strain Information** | | **Closest Match** | | | **QS^1^** | **QQ^2^** | | |
| --- | --- | --- | --- | --- | --- | --- | --- | --- |
| **Strain ID** | **NCBI Accession**  **No.** | **NCBI ID** | **NCBI Accession No.** | **% of Matching** | **Bio_MS^3^** | **3OC6-HSL** | **3OC8-HSL** | **3OC12-HSL** |
| **Bacteriodetes** | | | | | | | | |
| N035a | KC252709 | *Chryseobacterium indologenes* strain McR-1 | JF894157.1 | 99 | -- | -- | -- | + |
| N045 | KC252719 | *Chryseobacterium indologenes* strain McR-1 | JF894157.1 | 99 | -- | -- | -- | +++ |
| N046 | KC252720 | *Chryseobacterium indologenes* strain McR-1 | JF894157.1 | 99 | -- | -- | -- | +++ |
| N047 | KC252721 | *Chryseobacterium indologenes* strain McR-1 | JF894157.1 | 99 | -- | -- | -- | +++ |
| N048 | KC252722 | *Chryseobacterium indologenes* strain McR-1 | JF894157.1 | 99 | -- | -- | -- | +++ |
| R001 | KC252829 | *Chryseobacterium indologenes* strain McR-1 | JF894157.1 | 99 | -- | -- | -- | +++ |
| R002 | KC252830 | *Chryseobacterium indologenes* strain McR-1 | JF894157.1 | 99 | -- | -- | -- | +++ |
| R003 | KC252831 | *Chryseobacterium indologenes* strain McR-1 | JF894157.1 | 99 | -- | -- | -- | +++ |
| R004 | KC252832 | *Chryseobacterium indologenes* strain McR-1 | JF894157.1 | 99 | -- | -- | -- | +++ |
| R005b | KC252834 | *Chryseobacterium indologenes* strain McR-1 | JF894157.1 | 99 | -- | -- | -- | +++ |
| R059 | KC252889 | *Chryseobacterium indologenes* strain McR-1 | JF894157.1 | 99 | -- | -- | -- | +++ |
| R061 | KC252891 | *Chryseobacterium indologenes* strain McR-1 | JF894157.1 | 99 | -- | -- | -- | +++ |
| R062 | KC252892 | *Chryseobacterium indologenes* strain McR-1 | JF894157.1 | 99 | -- | -- | -- | +++ |
| R064 | KC252894 | *Chryseobacterium indologenes* strain McR-1 | JF894157.1 | 99 | -- | -- | -- | +++ |
| R065 | KC252895 | *Chryseobacterium indologenes* strain McR-1 | JF894157.1 | 99 | -- | -- | -- | +++ |
| R067b | KC252898 | *Chryseobacterium indologenes* strain McR-1 | JF894157.1 | 99 | -- | -- | -- | +++ |
| R068b | KC252902 | *Chryseobacterium indologenes* strain McR-1 | JF894157.1 | 99 | -- | -- | -- | +++ |
| R085 | KC252919 | *Chryseobacterium indologenes* strain McR-1 | JF894157.1 | 99 | -- | -- | -- | +++ |
| R048 | KC252877 | *Cloacibacterium normanense* strain tu29 | FJ544401.1 | 100 | -- | -- | -- | +++ |
| R091 | KC252925 | *Cloacibacterium normanense* strain CCUG 46293 | NR_042187.1 | 98 | -- | -- | -- | -- |
| N010* | KC252683 | *Dyadobacter fermentans* strain DSM 18053 | CP001619.1 | 87 | -- | -- | -- | +++ |
| R050* | KC252879 | *Dyadobacter fermentans* strain DSM 18053 | CP001619.1 | 87 | -- | -- | -- | +++ |
| R098* | KC252932 | *Dyadobacter fermentans* strain DSM 18053 | CP001619.1 | 87 | -- | -- | -- | +++ |
| N015b | KC252687 | *Elizabethkingia anophelis* strain 5.33 | EF426430.1 | 99 | -- | -- | -- | -- |
| R029a | KC252857 | *Elizabethkingia anophelis* strain 5.33 | EF426430.1 | 99 | -- | -- | -- | -- |

**Table S2.** Continued.

| **Strain Information** | | **Closest Match** | | | **QS^1^** | **QQ^2^** | | |
| --- | --- | --- | --- | --- | --- | --- | --- | --- |
| **Strain ID** | **NCBI Accession No.** | **NCBI ID** | **NCBI Accession No.** | **% of Matching** | **Bio_MS^3^** | **3OC6-HSL** | **3OC8-HSL** | **3OC12-HSL** |
| **Bacteriodetes** | | | | | | | | |
| R029b | KC252858 | *Elizabethkingia anophelis* strain 5.33 | EF426430.1 | 99 | -- | -- | -- | -- |
| R036N | KC252865 | *Elizabethkingia anophelis* strain 5.33 | EF426430.1 | 99 | -- | -- | -- | -- |
| N009 | KC252682 | *Flavobacterium banpakuense* strain 15F3 | GQ281770.1 | 97 | -- | -- | -- | +++ |
| P041b | KC252779 | *Flavobacterium banpakuense* strain 15F3 | GQ281770.1 | 97 | -- | -- | -- | +++ |
| R030N | KC252859 | *Flavobacterium* sp. NL124 | AB636296.1 | 99 | -- | -- | -- | -- |
| R041 | KC252870 | *Flavobacterium* sp. NL124 | AB636296.1 | 99 | -- | -- | -- | -- |
| R046 | KC252875 | *Flavobacterium* sp. NL124 | AB636296.1 | 99 | -- | -- | -- | +++ |
| N007N | KC252680 | *Pedobacter composti* strain TR6-06 | NR_041506.1 | 99 | -- | -- | -- | +++ |
| N013N | KC252685 | *Pedobacter composti* strain TR6-06 | NR_041506.1 | 99 | -- | -- | -- | +++ |
| R031 | KC252860 | *Pedobacter composti* strain TR6-06 | NR_041506.1 | 99 | -- | -- | -- | +++ |
| R032N | KC252861 | *Pedobacter composti* strain TR6-06 | NR_041506.1 | 99 | -- | -- | -- | -- |
| R033 | KC252862 | *Pedobacter composti* strain TR6-06 | NR_041506.1 | 99 | -- | -- | -- | +++ |
| R034N | KC252863 | *Pedobacter composti* strain TR6-06 | NR_041506.1 | 99 | -- | -- | -- | -- |
| R035N | KC252864 | *Pedobacter composti* strain TR6-06 | NR_041506.1 | 99 | -- | -- | -- | -- |
| R047N | KC252876 | *Pedobacter composti* strain TR6-06 | NR_041506.1 | 99 | -- | -- | -- | +++ |
| R056 | KC252886 | *Pedobacter composti* strain TR6-06 | NR_041506.1 | 99 | -- | -- | -- | + |
| R080N | KC252914 | *Pedobacter composti* strain TR6-06 | NR_041506.1 | 99 | -- | -- | -- | + |
| R090N | KC252924 | *Pedobacter composti* strain TR6-06 | NR_041506.1 | 99 | -- | -- | -- | +++ |
| R016a | KC252846 | *Sphingobacterium mizutaii* strain LMG 8340 | JF708889.1 | 100 | -- | -- | -- | +++ |
| R016b | KC252847 | *Sphingobacterium mizutaii* strain LMG 8340 | JF708889.1 | 100 | -- | -- | -- | +++ |
| P031 | KC252768 | *Sphingobacterium mizutaii* strain NBRC 14946 | JF708889.1 | 98 | -- | -- | -- | +++ |
| P004 | KC252742 | *Sphingobacterium multivorum* strain IAM14316 | NR_040953.1 | 99 | -- | -- | -- | -- |
| P044 | KC252784 | *Sphingobacterium multivorum* strain IAM14316 | NR_040953.1 | 99 | -- | -- | -- | + |
| P049 | KC252791 | *Sphingobacterium multivorum* strain IAM14316 | NR_040953.1 | 99 | -- | -- | -- | + |
| P057 | KC252798 | *Sphingobacterium multivorum* strain IAM14316 | NR_040953.1 | 99 | -- | -- | -- | -- |
| P066N | KC252807 | *Sphingobacterium multivorum* strain IAM14316 | NR_040953.1 | 99 | -- | -- | -- | -- |

**Table S2.** Continued.

| **Strain Information** | | **Closest Match** | | | **QS^1^** | **QQ^2^** | | |
| --- | --- | --- | --- | --- | --- | --- | --- | --- |
| **Strain ID** | **NCBI Accession No.** | **NCBI ID** | **NCBI Accession No.** | **% of Matching** | **Bio_MS^3^** | **3OC6-HSL** | **3OC8-HSL** | **3OC12-HSL** |
| **Bacteriodetes** | | | | | | | | |
| P084 | KC252822 | *Sphingobacterium multivorum* strain IAM14316 | NR_040953.1 | 99 | -- | -- | -- | + |
| P089 | KC252827 | *Sphingobacterium multivorum* strain DW-1 | EU240954.1 | 99 | -- | -- | -- | -- |
| **Firmicutes** | | | | | | | | |
| N059b | KC252732 | *Bacillus firmus* strain N12-3 | HM030743.1 | 99 | -- | -- | -- | + |
| A008b | KC252644 | *Bacillus megaterium* strain As-30 | JF895489.1 | 100 | -- | -- | -- | + |
| R054bN | KC252884 | *Bacillus niabensis* strain m10 | JF411233.1 | 99 | -- | -- | -- | -- |
| P048b | KC252790 | *Staphylococcus condimenti* strain F-2 | NR_029345.1 | 100 | -- | -- | -- | -- |
| S001N | KC252933 | *Staphylococcus haemolyticus* strain HNMCTR1 | JF775574.1 | 100 | -- | -- | -- | -- |
| S006N | KC252937 | *Staphylococcus haemolyticus* strain HNMCTR1 | JF775574.1 | 100 | -- | -- | -- | -- |
| S009b | KC252939 | *Staphylococcus haemolyticus* strain HNMCTR1 | JF775574.1 | 100 | -- | -- | -- | -- |
| S011b | KC252942 | *Staphylococcus haemolyticus* strain HNMCTR1 | JF775574.1 | 100 | -- | -- | -- | -- |
| **Fungi** | | | | | | | | |
| A028 | KC252943 | *Candida sojae* strain CBS 7871 | JQ647916.1 | 100 | -- | -- | -- | -- |
| N043N | KC252944 | *Candida sojae* strain CBS 7871 | JQ647916.1 | 100 | -- | -- | -- | -- |
| N052 | KC252945 | *Candida sojae* strain CBS 7871 | JQ647916.1 | 100 | -- | -- | -- | +++ |
| P030 | KC252946 | *Candida sojae* strain CBS 7871 | JQ647916.1 | 100 | -- | -- | -- | -- |
| P068 | KC252947 | *Candida sojae* strain CBS 7871 | JQ647916.1 | 100 | -- | -- | -- | -- |
| P077 | KC252948 | *Candida sojae* strain CBS 7871 | JQ647916.1 | 100 | -- | -- | -- | -- |
| P078 | KC252949 | *Candida sojae* strain CBS 7871 | JQ647916.1 | 100 | -- | -- | -- | -- |
| P091N | KC252950 | *Candida sojae* strain CBS 7871 | JQ647916.1 | 100 | -- | -- | -- | -- |
| P018 | KC252951 | *Candida tropicalis* isolate ypy06 | HQ412611.1 | 100 | -- | -- | -- | + |
| P033 | KC252952 | *Candida tropicalis* isolate ypy06 | HQ412611.1 | 100 | -- | -- | -- | + |
| P034N | KC252953 | *Candida tropicalis* isolate ypy06 | HQ412611.1 | 100 | -- | -- | -- | -- |
| P050 | KC252954 | *Candida tropicalis* isolate ypy06 | HQ412611.1 | 100 | -- | -- | -- | -- |
| N011 | KC252955 | *Cryptococcus curvatus* strain ATCC 10567 | EU266558.1 | 100 | -- | -- | -- | +++ |
| N023 | KC252956 | *Cryptococcus curvatus* strain ATCC 10567 | EU266558.1 | 100 | -- | -- | -- | +++ |

**Table S2.** Continued.

| **Strain Information** | | **Closest Match** | | | **QS^1^** | **QQ^2^** | | |
| --- | --- | --- | --- | --- | --- | --- | --- | --- |
| **Strain ID** | **NCBI Accession No.** | **NCBI ID** | **NCBI Accession No.** | **% of Matching** | **Bio_MS^3^** | **3OC6-HSL** | **3OC8-HSL** | **3OC12-HSL** |
| **Fungi** | | | | | | | | |
| N040 | KC252957 | *Cryptococcus curvatus* strain ATCC 10567 | EU266558.1 | 100 | -- | -- | -- | +++ |
| N051 | KC252958 | *Cryptococcus curvatus* strain ATCC 10567 | EU266558.1 | 100 | -- | -- | -- | +++ |
| R021 | KC252959 | *Cryptococcus curvatus* strain ATCC 10567 | EU266558.1 | 100 | -- | -- | -- | +++ |
| R022 | KC252960 | *Cryptococcus curvatus* strain ATCC 10567 | EU266558.1 | 100 | -- | -- | -- | +++ |
| S002 | KC252961 | *Cryptococcus curvatus* strain ATCC 10567 | EU266558.1 | 100 | -- | -- | -- | +++ |
| S008 | KC252962 | *Cryptococcus curvatus* strain ATCC 10567 | EU266558.1 | 100 | -- | -- | -- | +++ |
| S004a | KC252963 | *Meyerozyma guilliermondii* strain W56260-07 | HQ693808.1 | 100 | -- | -- | -- | + |
| S007 | KC252964 | *Meyerozyma guilliermondii* strain W56260-07 | HQ693808.1 | 100 | -- | -- | -- | + |
| R018 | KC252965 | *Trichosporon montevideense* isolate CLOA70 | GU299461.1 | 99 | -- | -- | + | +++ |

^1^QS – Quorum sensing assessed by both bioassays and LC-MS/MS: -- Negative; +++ Positive

^2^QQ – Quorum quenching: -- Negative; + Weak positive; ++ Medium positive; +++ Strong positive

^3^Bio_MS – QS was assessed by both bioassays (*E. coli* JBA357, *A. tumefaciens* A136 and *C. violaceum* CV026) and LC-MS/MS.

* Classification shown in Figure 4: R042 – Comamonadaceae bacterium; R051 – Xanthomonadaceae bacterium; N010, R050, R098 – Cytophagaceae bacterium.

All the experiments were independently repeated for three times.
