# Supplementary material for: Community quorum sensing signalling and quenching: microbial granular biofilm assembly
Source: NPJ Biofilms Microbiomes. 2015 May 27;1:15006–. doi: 10.1038/npjbiofilms.2015.6 (PMC5515215; doi:10.1038/npjbiofilms.2015.6)
Supplement: Supplementary Table S3 [file npjbiofilms20156-s3.doc]

**Table S3. Meta-rRNA studies: Statistics of microbiota data**

| **Week** | **V6 Sequences^1^** | **Unique V6 tag^2^** | **Simpson (1/D)** | **Shannon (H')** | **Shannon-Weaver (e^H'^)** |
| --- | --- | --- | --- | --- | --- |
| **1** | 8331 | 191 | 5.1 | 2.8 | 16.9 |
| **9** | 7958 | 227 | 10.0 | 3.2 | 24.7 |
| **13** | 7455 | 169 | 7.9 | 2.8 | 17.1 |
| **16** | 12794 | 215 | 6.1 | 2.7 | 14.8 |
| **30** | 8183 | 154 | 3.1 | 2.1 | 8.4 |
| **34** | 10232 | 147 | 4.8 | 2.3 | 9.5 |
| **40** | 9243 | 143 | 3.4 | 2.1 | 8.3 |
| **44** | 11030 | 156 | 5.1 | 2.2 | 9.2 |
| **48** | 10205 | 160 | 6.3 | 2.5 | 12.3 |
| **51** | 6981 | 132 | 5.4 | 2.2 | 9.4 |
| **59** | 4801 | 111 | 5.8 | 2.4 | 11.4 |
| **61** | 9713 | 159 | 5.8 | 2.4 | 11.4 |
| **65** | 10869 | 180 | 5.6 | 2.5 | 12.3 |
| **73** | 12269 | 171 | 3.8 | 2.3 | 10.2 |
| **77** | 12829 | 198 | 4.8 | 2.4 | 11.1 |
| **82** | 13270 | 184 | 3.9 | 2.1 | 7.9 |

^1^The hypervariable region 6 (V6) of the rRNA gene sequence is extracted from the total meta-rRNA sequences.

^2^Each different V6 tag is defined as one operational taxanomic unit (OTU), and most V6 tags represent OTUs at

taxomomic level between genus and species level.
