# Supplementary material for: Community quorum sensing signalling and quenching: microbial granular biofilm assembly
Source: NPJ Biofilms Microbiomes. 2015 May 27;1:15006–. doi: 10.1038/npjbiofilms.2015.6 (PMC5515215; doi:10.1038/npjbiofilms.2015.6)
Supplement: Supplementary Table S4 [file npjbiofilms20156-s4.doc]

| **Abundance ranking** | **V6 tag^1^** | **Identity^2^** | **Isolates matching to V6 tag** |
| --- | --- | --- | --- |
| 1 | GTGTTCAGGTTCTCTTGCGAGCACTCCCAAATC | Candidatus *Accumulibacter* (Genus) |  |
| 2 | GTCTCGGGGCTCCTTTCGGCACCCCCCATCTCT | NA |  |
| 3 | GTCTCGGGGCTCCTTGCGGCACCTCCCATCTCT | NA |  |
| 4 | GTGTTCAGGTTCTCTTGCGAGCACTCCCGAATC | Candidatus *Accumulibacter* (Genus) |  |
| 5 | GTGTTCTGGCTCCCGAAGGCACCCTCGCCTCTC | *Thauera* (Genus) | *Thauera* sp.- R052; R086 |
| 6 | GTCTCGGGGCTCCTTGCGGCACCGTCCATCTCT | Gammaproteobacteria (Class) |  |
| 7 | GTCTCGGGGTTCCTTGCGGCACTGCCCATCTCT | NA |  |
| 8 | GTGTCCAGGCTCCCTTTCGGGCACCAAGCCATC | Rhodocyclaceae (Family) |  |
| 9 | GTCTTGGGGCTCCTTTCGGCACCATCCATCTCT | NA |  |
| 10 | GTGTGGCGTCCAGCCGAACTGAAGTCCCAGGTC | NA |  |
| 11 | TGCTTTGTGTCCCGTAGGAAAATACCGTTTCCG | NA |  |
| 12 | GTGATCGCTGCCCGAAGGCTCGTTCCGCTTTCG | NA |  |
| 13 | GAGCACGCTGGTATTGCTACCTCGTCAGGCTTT | *Nitrospira* (Genus) |  |
| 14 | GTCACCGATCCAACTAAATGAAGGAGTCCTTTT | Alphaproteobacteria (Class) |  |
| 15 | GTCTCGGGGCTCCTTGCGGCACTGTCCATCTCT | Gammaproteobacteria (Class) |  |
| 16 | GTGTTCAGGCTCCCTTGCGGGCACTCCCGAATC | NA |  |
| 17 | GTGTCCAGGCTCTCTTTCGAGCACCAAGCCATC | Rhodocyclaceae (Family) |  |
| 18 | GTGCACGTTCCGGTCTTGCGACTGTGGCCCCGC | NA |  |
| 19 | GTGTTCTGGCTCCCGAAGGCACCCTCGGCTCTC | *Thauera* (Genus) |  |
| 20 | GTGTTCAAATTCTCTTTCGAGCACGATTCCATC | NA |  |
| 21 | GAGCACGCTGGTATTGCTACCTCGTTAGGCTTT | *Nitrospira* (Genus) |  |
| 22 | GTCTCGGGGCTCCTTGCGGCACCCCCCATCTCT | NA |  |
| 23 | GTGTTTAGATTTCCTTTCGGACACAATTTCATC | *Nitrosomonas* (Genus) |  |
| 24 | TACAGACTGTGTATTGCTACAAAGCCCTCTTTC | NA |  |
| 25 | ATATAGCGGCCCGAAGGCGCAAACATCTCTGCT | NA |  |
| 26 | GTCACCGGTCCAGCCGAACTGAAGGAAACGATC | Phyllobacteriaceae (Family) |  |
| 27 | TACAAACTGTGTATTGCTACAAAGCCCTCTTTC | NA |  |
| 28 | GTCTCTCGGCTCCCTCGTGAGAGGGCACCTCCG | NA |  |
| 29 | CGTAATATGTCCGAAGAAATATAGCTTTCACCA | Sphingobacteriales (Order) |  |

**Table S4.** The top 200 most abundant community member and the corresponding isolates.

**Table S4. Continued.**

| **Abundance ranking** | **V6 tag^1^** | **Identity^2^** | **Isolates matching to V6 tag** |
| --- | --- | --- | --- |
| 30 | GTGTCTCGGTTCTCTTTCGAGCACCTCCGCATC | NA |  |
| 31 | GTGCATGTTCCACCCTTGCGGGTGTGGCTCTGC | NA |  |
| 32 | GTGTGGTAGCCACCCGAAGTGAAGGACCAAGAT | NA |  |
| 33 | GTGTTCAAATTCCCTTTCGGGCACGATTTTATC | NA |  |
| 34 | GTGTCCAGATTCCCTTTCGGGCACCCCCACCTC | NA |  |
| 35 | GTGCTAGCTCCCCGAAGGGTCGTTCCGTTTTCA | Caldilineaceae (Family) |  |
| 36 | GTGCAAAGGCTCCCCGAAGGGCACCCCAACTTT | NA |  |
| 37 | GTACAAGCTCCCCGAAGGGTCACTCACCTTTCG | NA |  |
| 38 | GTGCATGTTCCGGTCTTGCGACTGTGGCTCTAC | NA |  |
| 39 | GTGCAAGCTCCCTTGCGGGTCGTTCACCTTTCG | Deltaproteobacteria (Class) |  |
| 40 | GTGTCCCGGTTCCCTTTCGGGCACCAAACCATC | NA |  |
| 41 | AGTTTCGTGCTCCGAAGAGAATCCTGCTTTCAC | NA |  |
| 42 | GTGTGACGTCCAGCCGAACTGAAAGACCGGTCT | *Amaricoccus* (Genus) |  |
| 43 | GTGTTACGGTTCTCTTTCGAGCACCAAGCCATC | Comamonadaceae (Family) | *Diaphorobacter nitroreducens* - R042 |
| 44 | GTGTTCAGGTTCTCTTGCGAGCACTCCCAAATA | NA |  |
| 45 | GTGCAAAGCGGGTATTGCTACCCTATCCGCCTT | NA |  |
| 46 | ATACAGGCAACCGACTTAACGGTTCATTCGACT | Chlorobi (Phylum) |  |
| 47 | TGATATCTGTCCTTGCGGACTAATCCATTTCTG | NA |  |
| 48 | GTGATAGCTTCCCGAAGGATCGTTCCACTTTCG | NA |  |
| 49 | AGTTTCGTGTCCCGAAGGAAAGACCCCTTTCAG | NA |  |
| 50 | GTGCAGGTCACACCCGAAGGTAATCAGCCAGCT | NA |  |
| 51 | GTGTTCCGGCTCCCTTTCGGGCACTCCCAAATC | NA |  |
| 52 | GTGTCTTAATTCCCTTTCGGGCACTTTTACATT | NA |  |
| 53 | AACTACAGGTTCTCCGAAGAGCACCCCGACCTT | Myxococcales (Order) |  |
| 54 | ATATAGCGGCCCGAAGGCGACCCTGTCTCCAGG | Deltaproteobacteria (Class) |  |
| 55 | GTGCAAAGCGGGTATTGCTACCCTATCCGGCTT | *Prosthecobacter* (Genus) |  |
| 56 | GTGTAACAGTTCACGGTTTCCCGTGAAACCCGA | NA |  |
| 57 | GTGCTAGCTCCCCGAAGGGTCGTTCCGCTTTCG | NA |  |
| 58 | GTCTCGGGGCTCCTTTCGGCACCACTCATCTCT | NA |  |

**Table S4.** Continued.

| **Abundance ranking** | **V6 tag^1^** | **Identity^2^** | **Isolates matching to V6 tag** |
| --- | --- | --- | --- |
| 59 | GTGTTCAGGTTCTCTTGCGAGATCGGAAGAGCG | NA |  |
| 60 | GTGTTCAGGTTCTCTTGCGAGATCGGAAGAGCA | NA |  |
| 61 | TGTTTCGTGTCCCGAAAGACTATACCGTTTCCG | NA |  |
| 62 | TGTTTCGTGTCCCGAAGGAAAGTCCCCTTTCAG | Bacteroidetes (Phylum) |  |
| 63 | GTGTTCCAGTTCCCTTTCGGGCACACCCAAATC | *Dechloromonas* (Genus) |  |
| 64 | GTGCATGTTCCACCCGAAGGCGTGGCTCGGCTT | Planctomycetaceae (Family) |  |
| 65 | GTGATGGTCCACCCGAAGTGACGACAAGCTTTC | NA |  |
| 66 | GTAATGGCTCCCCGAAGGGTCGTTCCGCTTTCG | NA |  |
| 67 | TGTAGAGAGTCCGAAGAAAGAAGTGTTTCCACC | NA |  |
| 68 | GTCTCTGGATTCCCTTGCGGGCACGCCGACTTT | NA |  |
| 69 | GTGTGGCGTCCGGCCGAACCGACCCCCAGGTCT | NA |  |
| 70 | TACTGGCAGTGTATTGCTACAAAAAGAGCTTTC | NA |  |
| 71 | GTGTTCAGGTTCTCTTGCGAGAGATCGGAAGAG | NA |  |
| 72 | TGTAGAGAGTCCGAAGAAATAAGTGTTTCCACC | NA |  |
| 73 | CGCACGCCGTCCGAAGAAGAGGATGTTTCCACC | NA |  |
| 74 | GTCTCGGGGCCCCTTTCGGCACCCCCCATCTCT | NA |  |
| 75 | TGTTTCGTGTCCCGAAGGAAAGTCTCCTTTCAG | NA |  |
| 76 | GTCACCGGTCCAGCCGAACTGAAGGAATCCATC | *Mesorhizobium* (Genus) |  |
| 77 | GCACAGGATTTCTCTTGCGAGTTGGCCGACGTT | NA |  |
| 78 | TGATACCGGTCCCGAAGGAAAATCAGCTTTCAC | NA |  |
| 79 | TGCTTTGTGTTCTATTGCTAGACCTACAGGCTT | NA |  |
| 80 | AGTTTCGCGTCATTTCTGAAAGACTCTTTTCGG | Bacteroidetes (Phylum) |  |
| 81 | GTGTTCAAATTCCTTTTCGGGCACGATTTTATC | NA |  |
| 82 | GTGTGGCGTCCGGCCGAACCGACTCCCCGCTCT | Alphaproteobacteria (Class) |  |
| 83 | GTGTTCAGGAGATCGGAAGAGCACACGTCTGAA | NA |  |
| 84 | GTGTTCAGGTTCTCTTGCGAGCACAGATCGGAA | NA |  |
| 85 | TGAGAACCGTCCGAAGAAAGGTCTGTTTCCAGA | NA |  |
| 86 | TACTATCCGTATATTGCTATAAAGACACCTTTC | Bacteroidetes (Phylum) |  |
| 87 | TGTTTCGTGTCATTGCTGACTGTAACATCTCTG | NA |  |

**Table S4.** Continued.

| **Abundance ranking** | **V6 tag^1^** | **Identity^2^** | **Isolates matching to V6 tag** |
| --- | --- | --- | --- |
| 88 | GTGTCCAGATTCCCTTTCGGGCACTCCCCAATC | Betaproteobacteria (Class) |  |
| 89 | GTGTTCCGATTCCCGAAGGCACTCCCGCATCTC | Gammaproteobacteria (Class) | *Frateuria* sp. - N027a; R011a |
| 89 | GTGTTCCGATTCCCGAAGGCACTCCCGCATCTC | Gammaproteobacteria (Class) | *Dokdonella* sp. - R051 |
| 90 | TCTCACTCGCTCCCACGAGGGGCACCCAGACAT | NA |  |
| 91 | CGATTGCCGTTCCCGAAGGAAACCCCGCTATTT | Acidobacteriaceae (Family) |  |
| 92 | TGCTCTTTGTTCTTGCGAAAAAATCCATCTCTG | NA |  |
| 93 | GTGCAGAGGCCCCGAAGGGAATTACTATCTCTA | NA |  |
| 94 | GTCTCGGGGCTCCTTTCGGCACCCCCCATCCCT | NA |  |
| 95 | TGCTCCATGTCCTTGCGGAAGAGTCCATCTCTG | Bacteroidetes (Phylum) |  |
| 96 | GTGCAGGTCACACCCGAAGGTAATCATCCGACT | Planctomycetales (Order) |  |
| 97 | GAGCACGCTGGTATTGCTACCTCGTTAAGCTTT | NA |  |
| 98 | GTGAGATCGGAAGAGCACACGTCTGAACTCCAG | NA |  |
| 99 | GTGTTCAGGTTCTCTTGCGAGCACTCCCGAATA | NA |  |
| 100 | GTGTTACGGCTCTCTTTCGAGCACTCCTGCGTC | *Ottowia* (Genus) |  |
| 101 | GTGCACGAGTCCCGAAGGACGCTACCATCTCTG | NA |  |
| 102 | GTGTTCAGGAGATCGGAAGAGCGTCGTGTAGGG | NA |  |
| 103 | GTGCAAAAGTCATAGATTACTCTATGAAAGATA | NA |  |
| 104 | GTGTTCAGGTTCTCAGATCGGAAGAGCACACGT | NA |  |
| 105 | GTATAGGCTCCCCGAAGGGTCGTTCCGCTTTCA | NA |  |
| 106 | GTGAGATCGGAAGAGCGTCGTGTAGGGAAAGAG | NA |  |
| 107 | GTGCAAACCGGTTATTGCTAACCTAGCTTACTT | NA |  |
| 108 | GTGTTCAGGTTCTCTTGCGAGCACTCCCAGATC | Betaproteobacteria (Class) |  |
| 109 | GTGTTGCAGCTCCCTTTCGGGCACTTTCTCATC | NA |  |
| 110 | GTCTCGGGGCTCCTTTCGGCAGATCGGAAGAGC | NA |  |
| 111 | GTCTCACGGTTCCCGAAGGCACCAATCCATCTC | Xanthomonadaceae (Family) | *Pseudoxanthomonas* sp. - P060; P079N |
| 111 | GTCTCACGGTTCCCGAAGGCACCAATCCATCTC | Xanthomonadaceae (Family) | *Lysobacter* sp. - R069; N012; R092; R097; R037; R082; R053; R044; R068a; R094; R072 |
| 112 | GTCTCACGATTCCCGAAGGCACCCCCGCATCTC | NA |  |

**Table S4.** Continued.

| **Abundance ranking** | **V6 tag^1^** | **Identity^2^** | **Isolates matching to V6 tag** |
| --- | --- | --- | --- |
| 113 | GTCTCGGGGCCCCTTGCGGCACCTCCCATCTCT | NA |  |
| 114 | GTGTTCAGAGATCGGAAGAGCACACGTCTGAAC | NA |  |
| 115 | GTCTTTGGGCTCCTTGCGGCACCCCCATATCTC | NA |  |
| 116 | GTGTCCAGGCTCCCTTGCGGGCACCCTCTCGTT | NA |  |
| 117 | GTCACCGGTCCAGCCGAACTGAAGGTTACCATC | *Mesorhizobium* (Genus) |  |
| 118 | GTGTCCAGGTTCCCTTGCGGGCACCCCCAGATC | NA |  |
| 119 | CTGCAAAAGCTCCCCGAAAGGCACCACCATATT | NA |  |
| 120 | GAGCACGCTGGTATTGCTACCTCGTAAGGCTTT | NA |  |
| 121 | GCGCAGGACCCTTACGGACCCCATATCTCTATG | NA |  |
| 122 | GTCTCGGGGCTCCTTTCGGCGCCCCCCATCTCT | NA |  |
| 123 | GTGTATCTGCCTATTGCTAGGGGTCCAAACTTT | NA |  |
| 124 | GTCACGGCTCCTTGCGGTCGGTCCCCTTTCGGT | Chloroflexi (Phylum) |  |
| 125 | TGTTTCGTGTAGTATTGCTACCAAAAAACCCTT | NA |  |
| 126 | GTGCAAACTCTCCTTGCGGAGTCGTCACCCTTT | NA |  |
| 127 | GTGCGGCAGCCTCGAAGGCCTCCGATCTTTCGA | Verrucomicrobia (Phylum) |  |
| 128 | GTCTCGGGGCTCCTTTCGGCCCCCCCCATCTCT | NA |  |
| 129 | GTGTTCAGAGATCGGAAGAGCGTCGTGTAGGGA | NA |  |
| 130 | CGCATCGTGCCCCGAAGGGAGATCGGCTTTCAC | NA |  |
| 131 | GTGATAGTCCACCCGAAGTGACGACAAGCTTTC | *Verrucomicrobium* (Genus) |  |
| 132 | GTGTCCAGGCTCTCTTTCGAGCACGAATCCATC | *Zoogloea* (Genus) |  |
| 133 | GTAGACCAGCCCCGAAGGGAAGGATACTTTCAT | NA |  |
| 134 | GTGTGGCATCCAGCCGAACTGAAAGGACCATCT | *Methylophilus* (Genus) |  |
| 135 | GTGTGGTATCCAGCCGAACTGAAAGGACCATCT | *Rhodobacter* (Genus) |  |
| 136 | GTCACCGGTCCAGCCGAACTGAAGGATTCCATC | *Mesorhizobium* (Genus) |  |
| 137 | GTGTGGCGTCCAGCCGAACTGAAGGAGCAGGTC | NA |  |
| 138 | GTCTCGGGGCTCCTTTCGGCACCCCCCATCTCC | NA |  |
| 139 | GTGTTGTATCCAGCCGAACTGAAGGAACCATCT | Rhodobacteraceae (Family) | *Rhodobacter* sp. - R087 |
| 140 | GTGTTCAGGTTCTCAGATCGGAAGAGCGTCGTG | NA |  |
| 141 | TGAAAGACGTCCGAAGAAATATCTGTTTCCAAA | NA | *Flavobacterium* sp. - R041; R046; R030N |

**Table S4.** Continued.

| **Abundance ranking** | **V6 tag^1^** | **Identity^2^** | **Isolates matching to V6 tag** |
| --- | --- | --- | --- |
| 142 | GTGTTCCCGCCAGCCGAACTGAAGGATTCCATC | Rhizobiales (Order) |  |
| 143 | GTTTAAACTCCCTTGCGGGTCGATCCAATTTCT | NA |  |
| 144 | GTGTGCAGGTCACCGAAGTGAAGAGATCCGTCT | *Devosia* (Genus) |  |
| 145 | GTCTCAGTGTTCCCGAAGGCACCAATCCATCTC | Gammaproteobacteria (Class) | *Thermomonas* sp. - R038; R039N |
| 146 | GTCACCGCGCCTCCGAAGAGGACCATCGATCTC | NA |  |
| 147 | GTGCTGGTTCCGGTCTTGCGACTGTGGCTCTAC | NA |  |
| 148 | GTGCACGTTCCATTCTTGCGATTGTCATCGATG | NA |  |
| 149 | GTGTTACGGTTCCCGAAGGCACAATCTCATCTC | *Conchiformibius* (Genus) |  |
| 150 | GTCTCGGGGCTCCTTGCGGCCCCTCCCATCTCT | NA |  |
| 151 | GTGTTCAGGTTCTCTTGCGAGCACTCCCAAGAT | NA |  |
| 152 | GTGCATGTTCCACCCGAAGGCGTGGCTCTGCTT | Planctomycetales (Order) |  |
| 153 | GTGTTCAGGTTCTCTTGCGAGCAGATCGGAAGA | NA |  |
| 154 | GTGTCCAGGCTCCCTTTCGGAGATCGGAAGAGC | NA |  |
| 155 | GTGTGGCGTCCAGCCGAACTGAAGTCCTAGGTC | NA |  |
| 156 | GGTCTAGCACCCTCGAAGGACGTCCCATTTCTG | NA |  |
| 157 | GTGTCCAGGCTCCCTTTCGGGCACCAAGAGATC | NA |  |
| 158 | TGTTTCGTGTCCCGAAGGAAAGTTCCCTTTCAG | NA |  |
| 159 | GTTTTCAAGTTCCCTTACGGGCACTCCCGCATT | NA |  |
| 160 | GTCTCGGGGCTCCTTTCGAGATCGGAAGAGCGT | NA |  |
| 161 | GTGCAAAGTGGGTATTGCTACCCTATCCGCCTT | NA |  |
| 162 | GTGCACGCTCCCCGAAGGGTCGTTCCGCTTTCG | Anaerolineaceae (Family) |  |
| 163 | GTGCTTGTTCCACTCTTGCGAGCGTGGCTCCTG | NA |  |
| 164 | GCGTTCAGGTTCTCTTGCGAGCACTCCCAAATC | NA |  |
| 165 | GTGCACGTTCCACCCGAAGGCGTGGCTCTCCTT | *Planctomyces* (Genus) |  |
| 166 | TGCTACCTCTCCATTGCTGGAACGCCATCTTTC | NA |  |
| 167 | TGTTTCGTGTCCCGAAGGAAAAACGGCTTTCAC | Sphingobacteria (Class) |  |
| 168 | GTGTTGCAGTTCTCTTTCGAGCACTTTCTCATC | NA |  |
| 169 | GTGCCCAGATTCCTTGCGGCACTCCCACATCTC | *Azonexus* (Genus) |  |
| 170 | GTCTCGGGGCTCCTTTCAGATCGGAAGAGCACA | NA |  |

**Table S4.** Continued.

| **Abundance ranking** | **V6 tag^1^** | **Identity^2^** | **Isolates matching to V6 tag** |
| --- | --- | --- | --- |
| 171 | GTCTCGGGGCTCCTTGCGGCACCTCCCAGATCG | NA |  |
| 172 | GTCTCTCGATTCCTTCTTGCGAAGGCACCCCAC | NA |  |
| 173 | GTGCAAGCTGGTATTGCTACCTCGTCACCCTTT | NA |  |
| 174 | GTCACCGGTCCAGCCGAACTGAAGGGATCCATC | Phyllobacteriaceae (Family) |  |
| 175 | GTCTCGGGGCTCCTTTCGAGATCGGAAGAGCAC | NA |  |
| 176 | GTGCACGTTCCAGCCGAAGCTGTGGCCCCGCTT | Planctomycetaceae (Family) |  |
| 177 | GTGTGGGATCCGGCCGAACCGACAACAATCATC | NA |  |
| 178 | GTGTCCAGGCTCCCTTTCGGGCACCAAGCAGAT | NA |  |
| 179 | GTGTTCAGGTTCTCTTGCGAGCCCTCCCAAATC | NA |  |
| 180 | GTGTTCCAGCTCCCTTTCGGGCACTCCCAAATC | Rhodocyclaceae (Family) |  |
| 181 | TGCTCCTTGTTCTTGCGAAAAAATCCATCTCTG | Cytophagales (Order) |  |
| 182 | GTGTCCTGTCCGGCCGAACCGACAGACCCGGTC | *Defluviicoccus* (Genus) |  |
| 183 | GTGTTCAGGCTCCCTTGCGGGCACTCCCAAATC | NA |  |
| 184 | GTGCACGTTGTACCCGAAGGCCTGGCTCCTGTT | Planctomycetales (Order) |  |
| 185 | GTGTTCAGGTTCTCTTGCAGATCGGAAGAGCAC | NA |  |
| 186 | GTCCCGGGGCTCCTTTCGGCACCCCCCATCTCT | NA |  |
| 187 | GTCTCGGGGCTCCTTGCGGCACCTCCCCTCTCT | NA |  |
| 188 | GTCTCGGGGCTCCTTGCGGCGCCTCCCATCTCT | NA |  |
| 189 | TGGAATCAGCCCCAAAGGGCGATCACCTTTCAG | NA |  |
| 190 | GTGTTCTCGCCAGCCGAACTGAAGAAAGTCATC | NA |  |
| 191 | GTGTTCAAGATCGGAAGAGCGTCGTGTAGGGAA | NA |  |
| 192 | GTGTCCACGTCCCCGAAGGGAAGAAATCCATCT | NA |  |
| 193 | GTCTCGGGGCTCCTTTCAGATCGGAAGAGCGTC | NA |  |
| 194 | GTCTCGGGGCTCCTCGCGGCACCGCCCATCTCT | NA |  |
| 195 | GTGCTTGTTCCACCCAGAGGGTGTGGCTCCTCT | NA |  |
| 196 | GTATACCGACCTTGCGGGGAGAACATTTCTGAA | *Frigoribacterium* (Genus) |  |
| 197 | GTGTTCAGGTTCTCTTAGATCGGAAGAGCGTCG | NA |  |
| 198 | GTCTCGGGGCTCCTTTCGGCACCCCCATCTCTG | NA |  |
| 199 | GTGTCCAGGCTCTCTTTCGAGCACCAAACCATC | *Thiomonas* (Genus) |  |

**Table S4.** Continued.

| **Abundance ranking** | **V6 tag^1^** | **Identity^2^** | **Isolates matching to V6 tag** |
| --- | --- | --- | --- |
| 200 | GTCTCGGGGCTCCTTTCGGCACCCCAGATCGGA | NA |  |
| >200 | GTCTCAATGTTCCCGAAGGCACCAATCTATCTC | *Pseudomonas* (Genus) | *Pseudomonas veronii* - P080; |
| >200 | GTGTGCGATCCAGCCGAACTGAAGGAACCATCT | *Rhodobacter* (Genus) | *Rhodobacter* sp. - R077N;N020; |
| >200 | GTGTTCCGGCCAGCCGAACTGAAGAAAGGCATC | *Bosea* (Genus) | *Bosea thiooxidans* - N014;N026;N008; *Bosea* sp. - R093N;R096; |

^1^Each different V6 tag is defined as one operational taxanomic unit (OTU), and most V6 tags represent OTUs at taxomomic level between genus and species

level.

^2^Abbrevation: NA – Not applicable. Unclassified based on the taxonomy classification pipeline stated in Methods.
