# Supplementary material for: Community quorum sensing signalling and quenching: microbial granular biofilm assembly
Source: NPJ Biofilms Microbiomes. 2015 May 27;1:15006–. doi: 10.1038/npjbiofilms.2015.6 (PMC5515215; doi:10.1038/npjbiofilms.2015.6)
Supplement: Supplementary Table S5 [file npjbiofilms20156-s5.doc]

| **Strain** | **Closest Match** | **C4-HSL** | **C6-HSL** | **3OC6-HSL** | **C7-HSL** | **C8-HSL** | **3OC8-HSL** | **C10-HSL** | **3OC10-HSL** | **C12-HSL** | **3OC12-HSL** | **3OHC12-HSL** | **C14-HSL** | **3OC14-HSL** |
| --- | --- | --- | --- | --- | --- | --- | --- | --- | --- | --- | --- | --- | --- | --- |
| **Alpha-proteobacteria** | | | | | | | | | | | | | | |
| N065 | *Rhizobium borbori* |  |  |  |  |  |  |  |  |  |  |  |  |  |
| R055 | *Rhizobium borbori* |  |  |  |  |  |  |  |  |  |  |  |  |  |
| R023 | *Rhodobacter maris* |  |  |  |  |  |  |  |  |  |  |  |  |  |
| R074 | *Rhodobacter maris* |  |  |  |  |  |  |  |  |  |  |  |  |  |
| R076N | *Rhodobacter maris* |  |  |  |  |  |  |  |  |  |  |  |  |  |
| S009a | *Rhodobacter maris* |  |  |  |  |  |  |  |  |  |  |  |  |  |
| S011a | *Rhodobacter maris* |  |  |  |  |  |  |  |  |  |  |  |  |  |
| N016 | *Shinella fusca* |  |  |  |  |  |  |  |  |  |  |  |  |  |
| R054a | *Sphingomonas* sp. |  |  |  |  |  |  |  |  |  |  |  |  |  |
| R060 | *Sphingomonas* sp. |  |  |  |  |  |  |  |  |  |  |  |  |  |
| **Beta-proteobacteria** | | | | | | | | | | | | | | |
| R081 | *Acidovorax facilis* |  |  |  |  |  |  |  |  |  |  |  |  |  |
| **Gamma-proteobacteria** | | | | | | | | | | | | | | |
| N027a | *Frateuria* sp. |  |  |  |  |  |  |  |  |  |  |  |  |  |
| R011a | *Frateuria* sp. |  |  |  |  |  |  |  |  |  |  |  |  |  |
| R037 | *Lysobacter brunescens* |  |  |  |  |  |  |  |  |  |  |  |  |  |
| R053 | *Lysobacter brunescens* |  |  |  |  |  |  |  |  |  |  |  |  |  |
| R092 | *Lysobacter brunescens* |  |  |  |  |  |  |  |  |  |  |  |  |  |
| R067d | *Pantoea stewartii* |  |  |  |  |  |  |  |  |  |  |  |  |  |
| N025 | *Stenotrophomonas* sp. |  |  |  |  |  |  |  |  |  |  |  |  |  |
| N064 | *Stenotrophomonas* sp. |  |  |  |  |  |  |  |  |  |  |  |  |  |
| P026NR | *Stenotrophomonas* sp. |  |  |  |  |  |  |  |  |  |  |  |  |  |
| P026NS | *Stenotrophomonas* sp. |  |  |  |  |  |  |  |  |  |  |  |  |  |
| P088 | *Stenotrophomonas* sp. |  |  |  |  |  |  |  |  |  |  |  |  |  |

**Table S5.** LC-MSMS profiling of AHLs produced by the representative isolates cultured in LB5 and R2A media.

Detected by LC-MSMS

Undetectable by LC-MSMS
