# Supplementary figures and images for: Community quorum sensing signalling and quenching: microbial granular biofilm assembly
Source: NPJ Biofilms Microbiomes. 2015 May 27;1:15006–. doi: 10.1038/npjbiofilms.2015.6 (PMC5515215; doi:10.1038/npjbiofilms.2015.6)

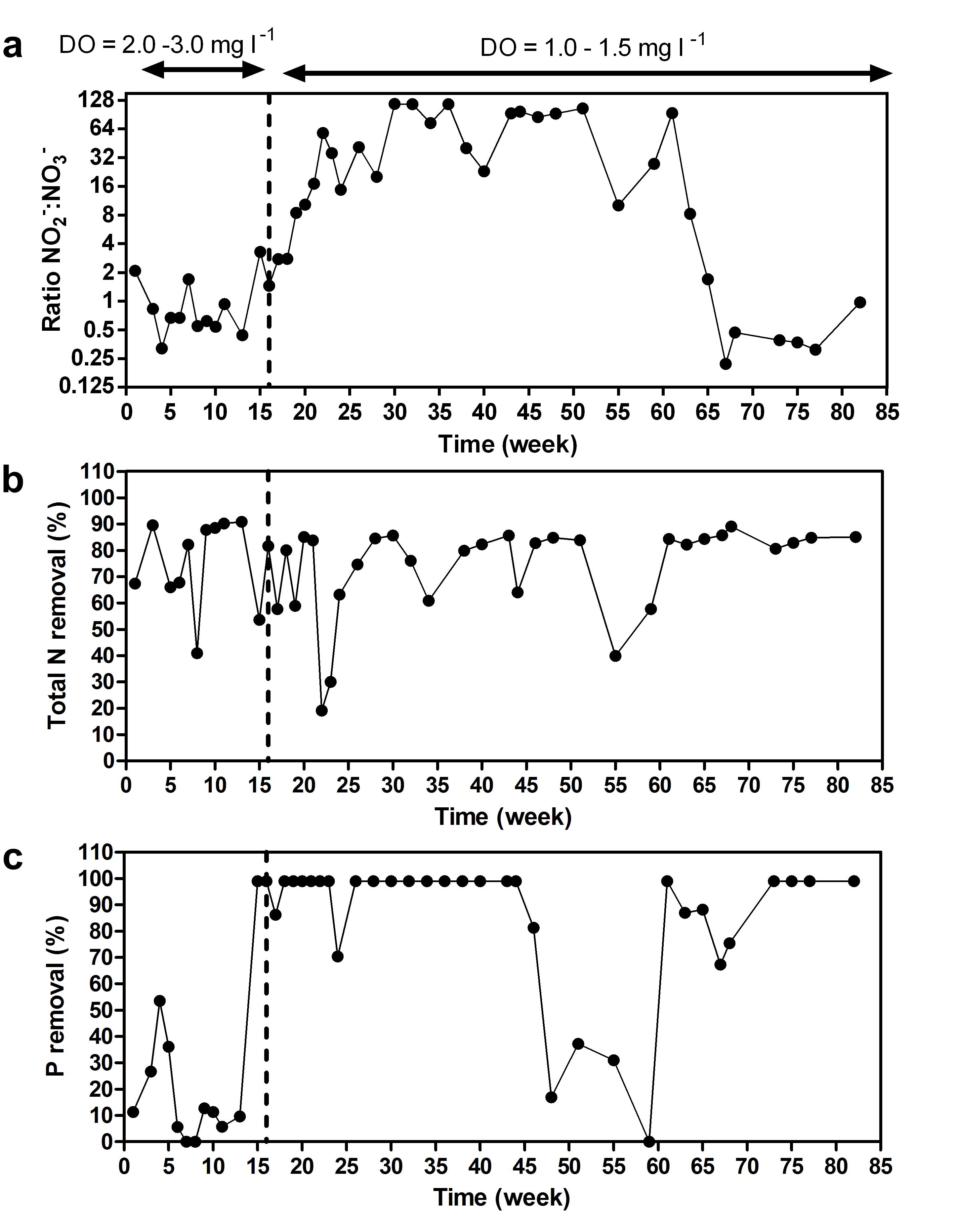

Supplement: Supplementary Figure S1 [file npjbiofilms20156-s6.jpg]

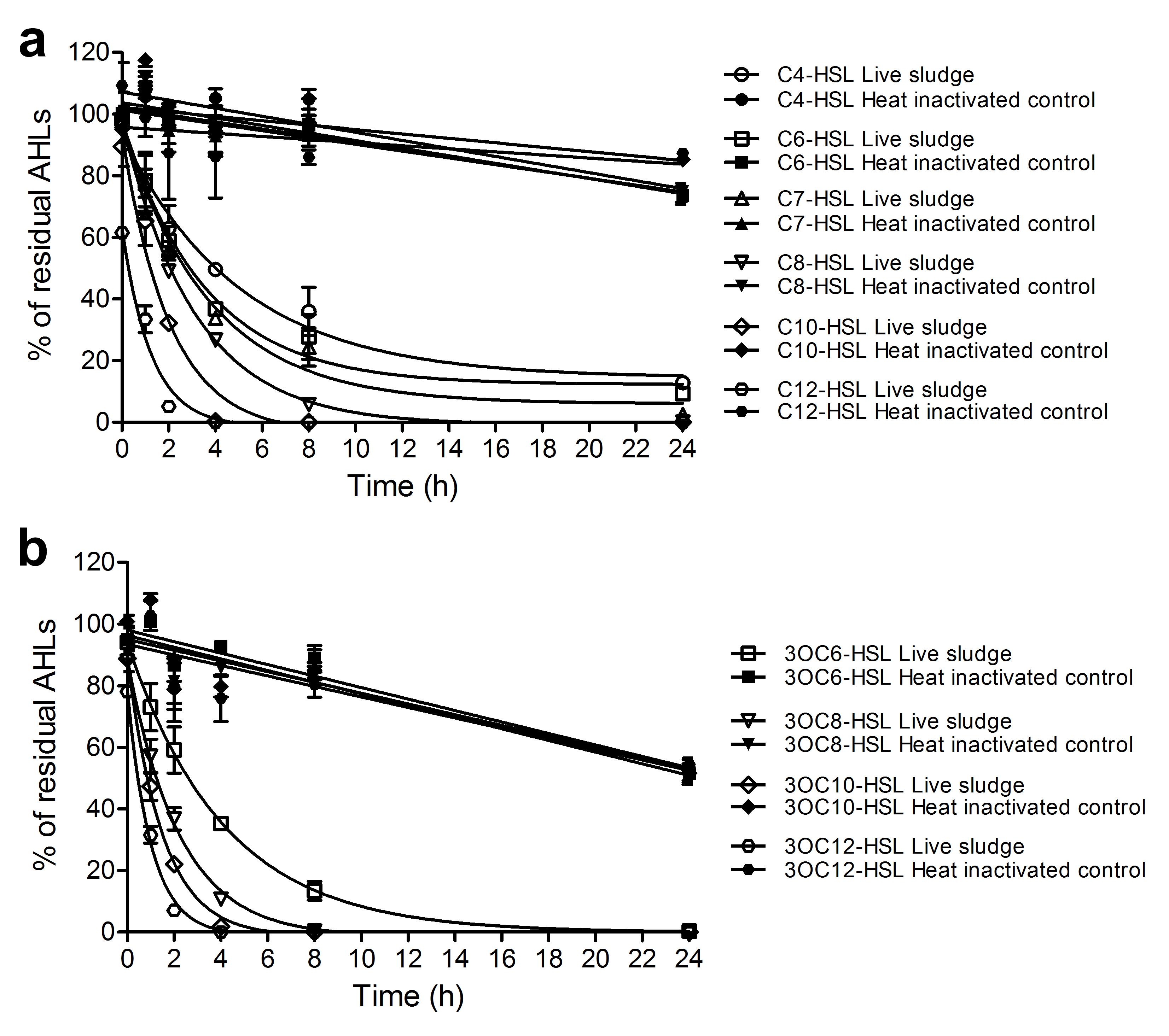

Supplement: Supplementary Figure S2 [file npjbiofilms20156-s7.jpg]

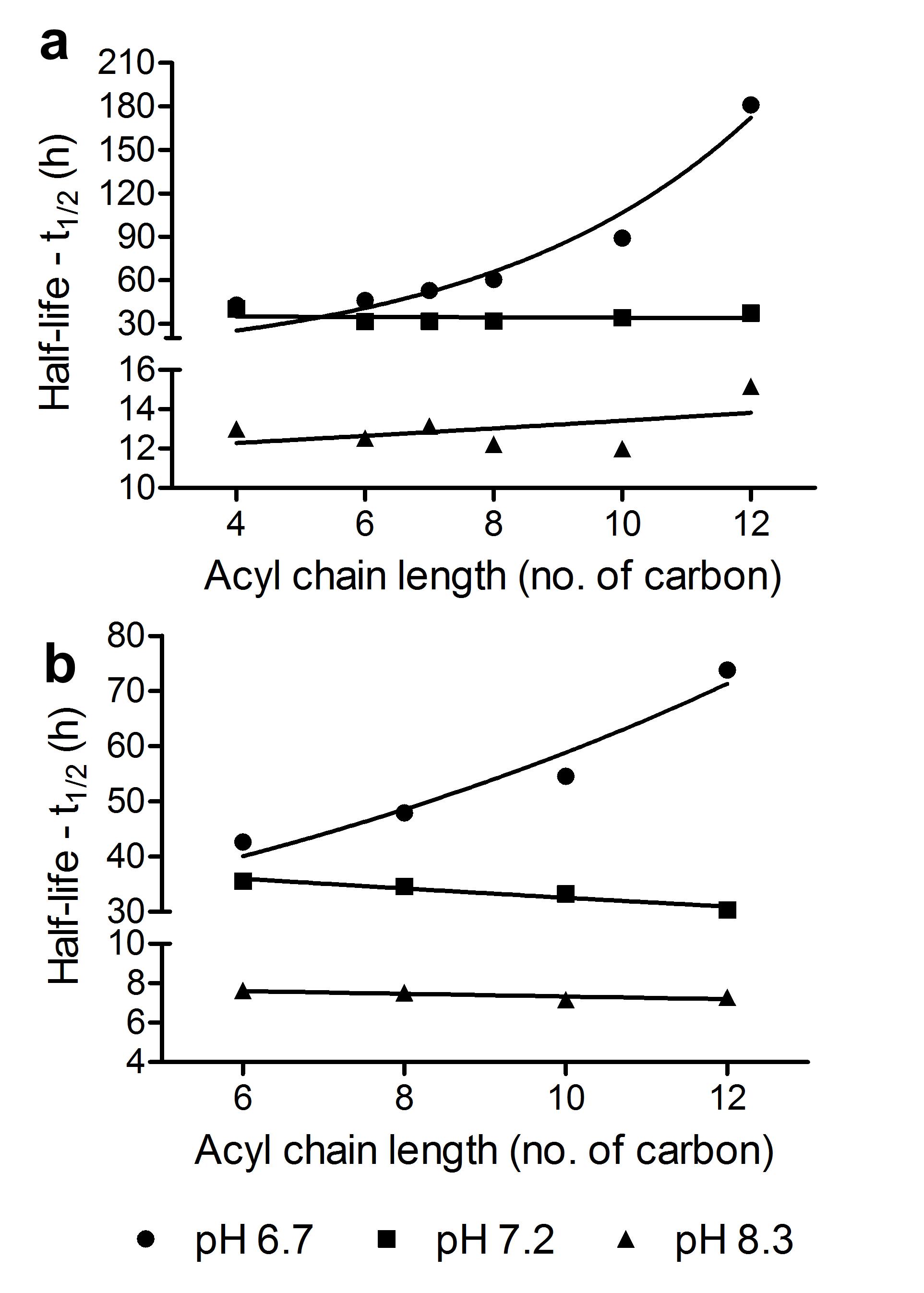

Supplement: Supplementary Figure S3 [file npjbiofilms20156-s8.jpg]

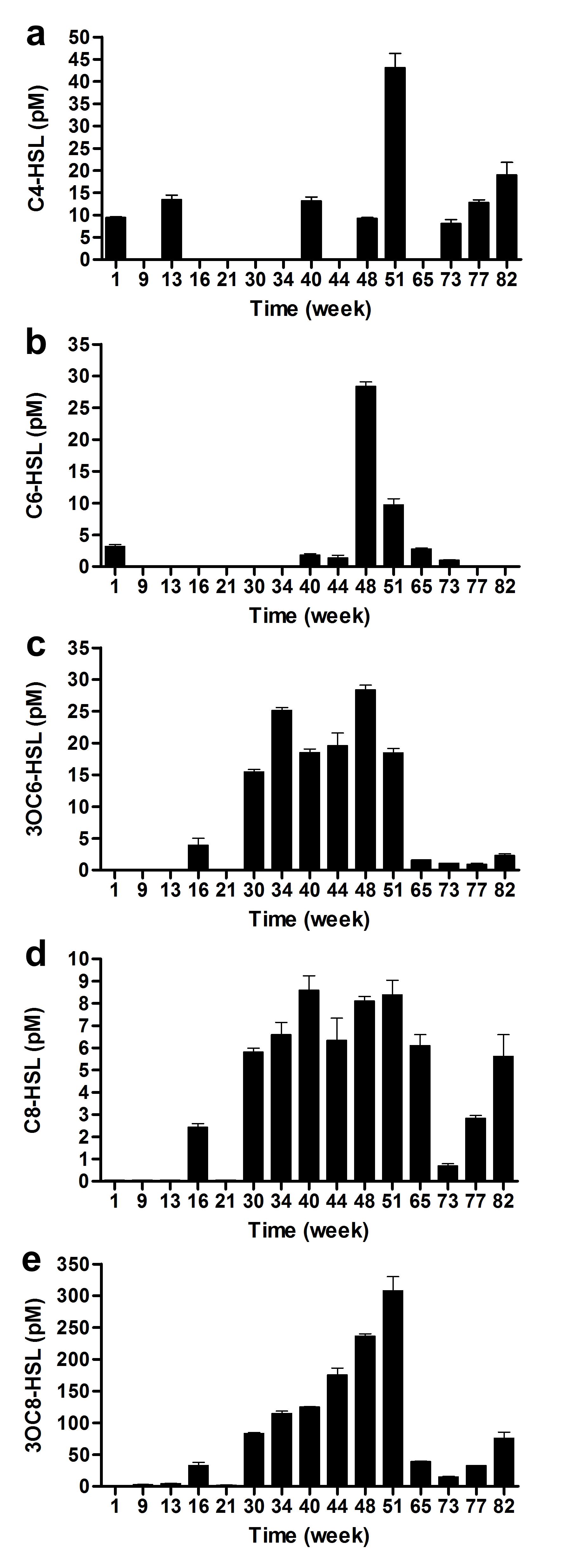

Supplement: Supplementary Figure S4 [file npjbiofilms20156-s9.jpg]

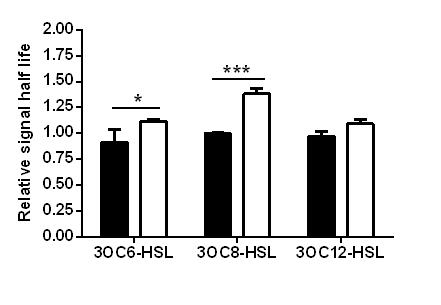

Supplement: Supplementary Figure S5 [file npjbiofilms20156-s10.jpg]

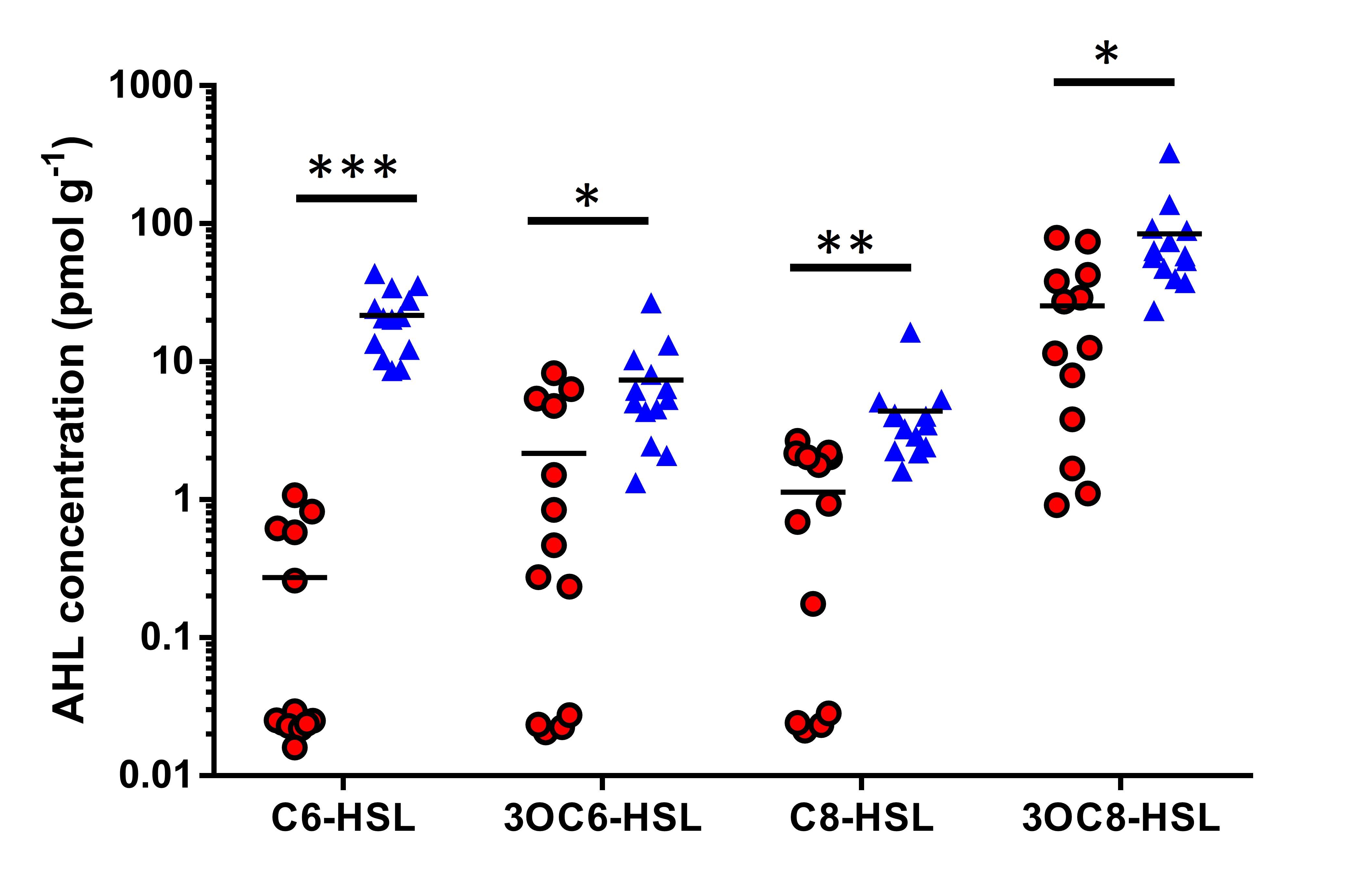

Supplement: Supplementary Figure S6 [file npjbiofilms20156-s11.jpg]
